# Association between pre-birth waist circumference and neonatal size and health in mother-newborn pairs in Lausanne, 1917–1921

**DOI:** 10.1371/journal.pone.0352179

**Published:** 2026-07-13

**Authors:** Rosa Hinselmann, Katarina L. Matthes, Kaspar Staub, Mathilde Le Vu

**Affiliations:** 1 Institute of Evolutionary Medicine, University of Zurich, Zurich, Switzerland; 2 Institut de recherche en santé, environnement et travail, Rennes, France; University of Rwanda College of Medicine and Health Sciences, RWANDA

## Abstract

**Background:**

Maternal anthropometric dimensions influence neonatal size and survival, yet waist circumference measured shortly before delivery remains little studied. Using historical maternity records from Lausanne, Switzerland (1917–1921), we examined associations between maternal waist circumference and neonatal anthropometry and perinatal health outcomes.

**Data and Methods:**

We analyzed 4,448 singleton births recorded at the Lausanne maternity hospital. Maternal waist circumference measured shortly before delivery was categorized as <80, 80–95, 95–110, and >110 cm. Dependent variables included birth weight, length, head circumference, ponderal index, preterm birth, low birth weight, macrosomia, microcephaly, stillbirth, and early neonatal mortality within five days. Missing data were handled using multiple imputation. Associations were estimated using generalized linear models adjusted for maternal age, gravidity, civil status, residence, infection during pregnancy, birth year and season, and infant sex.

**Results:**

Mean maternal waist circumference was 94.9 cm. Compared with the 80–95 cm reference group, waist circumference <80 cm was associated with lower birth weight (–575 g) and higher risks of low birth weight, preterm birth, microcephaly, stillbirth, and early neonatal death. Conversely, waist circumference >110 cm was associated with higher birth weight (+623 g) and substantially higher risk of macrosomia, but not higher infant mortality.

**Conclusion:**

In this historical cohort, maternal waist circumference before delivery was strongly associated with neonatal size and adverse outcomes. Small circumference was associated with lower birth weight, prematurity, and a higher risk of mortality, whereas a large circumference was associated with macrosomia. This dataset shows that pre-delivery waist circumference was associated with perinatal risk, offering insights relevant to historical populations.

## 1. Introduction

Pregnancy and childbirth are complex physiological processes that pose substantial health risks to both mother and newborn. Since the beginning of the 20th century maternal and/or infant mortality have decreased sharply thanks to several factors, including advances in clinical medicine, perinatal care, access to health care in general, and increased standards of living [1]. Although global maternal deaths declined by 44% between 1990 and 2015, there is still a large gap between low- and high-income countries in maternal and newborn mortality and morbidity [2].

External maternal anthropometric measures, such as maternal body height, as well as body weight, Body Mass Index (BMI), and body fat distribution, are associated with fetal health and growth during pregnancy [3]. BMI does not necessarily reflect body shape in pregnancy, additional measures to identify women at risk for adverse perinatal events may be useful for outcome prediction, timed intervention, and patient counseling [4]. There are several studies that have measured maternal waist circumference (WC) to predict neonatal size or pregnancy outcomes, but in these cases the measurements were not taken immediately before delivery but earlier during pregnancy. However, these studies show that larger WC in early pregnancy (10–13 weeks) seems to be associated with larger newborns [5]. In addition to BMI and WC, other maternal measures in use that better depict body fat distribution are the waist-to-hip-ratio (WHR) [4,6] and waist-to-height-ratio (WHtR) [7]. For example, it has been shown that women with elevated pre pregnancy WHR are more likely to deliver macrosomia newborns (birth weight >4,000 grams) [8]. In addition, the size of the mother’s pelvis in relation to the fetal head circumference (cephalo-pelvic disproportion) affects whether or not a vaginal birth is possible [9].

Since 1990, infant survival after birth has increased globally, but as with maternal complications of childbirth, there are large global disparities today [10]. The countries with the highest neonatal mortality rates (death in the first 28 days of life) reached 24 neonatal deaths per 1,000 live births in 2015, which is still far away from Goal 1 set by the United Nations for 2030, of 12 deaths per 1,000 live births [11]. Globally, the neonatal mortality rate is declining more slowly than the mortality rate for children aged 1 month to 5 years. Therefore, neonatal health and how to address it through clinical assessments remains a great concern [12]. Low birth weight (LBW, birth weight <2,500 grams) can result from restricted fetal growth or shorter gestational duration, and is used as an indicator of in utero malnutrition [13]. In addition, being born with a LBW can have lifelong consequences and can be associated with poor childhood growth and higher incidence of adult diseases such as type 2 diabetes, hypertension and cardiovascular disease [14]. Maternal and neonatal factors commonly associated with birth weight include gestational age, infant sex, gravidity, maternal BMI, maternal body height, gestational weight gain, maternal infections, ethnicity, lifestyle factors such as smoking, and maternal age, and socioeconomic variables such as maternal education level and income [3].

In high-income countries on the one hand, the incidence of macrosomia is currently increasing along with the incidence of maternal obesity and overweight [15]. Macrosomia has adverse clinical and social consequences for both mother and infant (higher rates of stillbirth, shoulder dystocia, Intensive Care Unit (ICU) admission, cesarean section, obstetric injury, etc.) [8]. In this context, maternal obesity in pregnancy is a prominent risk factor for gestational diabetes mellitus and thus also fetal macrosomia [16]. The interplay of maternal obesity and macrosomia has stimulated a substantial body of research on prenatal and early pregnancy predictors of fetal macrosomia [8]. In low-income countries on the other hand, malnutrition of pregnant women is still very common [17]. Maternal undernutrition during pregnancy results in placental and fetal growth restriction with increased risk of stillbirth, neonatal mortality and morbidity, inhibited growth and cognitive development, and chronic diseases later in life [13]. Mothers in deprived socio-economic conditions frequently have LBW infants. In those settings, the infant’s LBW stems primarily from the mother’s poor nutrition and health over a long period of time, including during pregnancy, the high prevalence of infections, or from pregnancy complications underpinned by poverty [18]. Physically demanding work during pregnancy also contributes to poor fetal growth [18].

Today’s high-income countries have a high prevalence of obesity and overweight [19]. Depending on studied populations, around 27–38 percent of women in Switzerland are overweight or obese today [20]. However, this was not always the case; these countries evolved during the 20th century from a population with a high prevalence of malnutrition and underweight to the current situation [21]. In Swiss cities 100 years ago, women were by average ca. 10 cm shorter than today, obesity was restricted to single cases and malnutrition was much more common, infectious diseases were more important than chronic diseases, hard physical work was common during pregnancy, and Gross Domestic Product (GDP) per capita was much lower than nowadays [22].

In this paper, we focus on a historical context in the Swiss city of Lausanne during the years 1917–1921. At that time, physicians documented the deliveries in great detail, and these data have been preserved in archives until today. Our main goal is to identify maternal anthropometric factors associated with a wide range of poor neonatal health outcomes in this historical setting. Specifically, we focus on maternal WC measured just before delivery, and how it was associated with infant birth weight and size, gestational age, and risks of mortality.

## 2. Data and methods

The time around 1920 was a period of transition following the difficult years at the end of the First World War in Europe and the outbreak of the 1918–20 influenza pandemic known as the “Spanish flu.” Although not directly involved in the war, Switzerland experienced significant economic disruption. While socioeconomic conditions in the final years of the war (1917 and 1918) did not rise to the level of causing famine, they did have noticeable negative effects on overall nutrition and especially neonatal health [23]. These effects were probably exacerbated by the influenza pandemic of 1918–20, which affected about 50–60% of the total population in several waves [24].

In a wider historical perspective, Switzerland had become one of Europe’s wealthiest countries in terms of GDP by the early 20th century [22]. From the late 19th century onward, per capita income rose steadily, accompanied by a marked expansion of urban areas. Using average height as a measure of standard of living, Switzerland at the beginning of the 20th century roughly mirrored the average height observed in Central Europe [25]. Consistent with data from comparable sources referenced in this study, women born in Switzerland between 1890 and 1899 had an average height of 158–162 cm [26].

This article examines a large city in southwestern Switzerland, Lausanne. Between 1890 and 1925, the population of Lausanne, primarily a French-speaking urban center, doubled from roughly 34,000 to nearly 70,000. Over these years, several measures of living standards showed clear improvement. Mortality rates, including those for infants and children, fell, and life expectancy rose markedly: a child born in the 1920s could expect to live around 59.1 years [27]. Infectious diseases diminished as part of the epidemiological transition and as sanitation and hygiene practices improved [28].

The study draws on the carefully kept and preserved birth records from Lausanne’s maternity hospital in the early 20th century. This institution, the sole maternity hospital in the canton of Vaud (where Lausanne is situated), served women across a range of socio-economic backgrounds, including those experiencing complicated deliveries. As the number and proportion of hospital births grew, the facility underwent expansion between 1900 and 1920. Lausanne Hospital became a university hospital in 1890, and by 1920 it accounted for 66% of all births in the city [29].

### 2.1. Ethics, data transcription, variable selection and data preparation

This study uses a data set that has already been described in other studies focusing on different research questions [9,30,31]. The larger research project, of which this study was a part, was approved by the Zurich Ethic Commission (BASEC Number 2021–00628). Data collection took place from 1 January 2022–31 August 2024. In accordance with the ethics approval, the team of authors had access to potentially identifying information from the retrospective archival data, which was over 100 years old, during the data collection phase. However, after data collection, the data set was completely anonymized. Deliveries contained in the books KVIII e 227–255, covering the years 1917–1921, were transcribed [32]. Events related to abortions or multiple births, or for which records were incomplete were not transcribed.

#### Lack of historical measurement records.

Despite extensive archival research, it has not been possible to locate the anthropometric measurement protocols from that period at this hospital. This applies not only to the WC and other maternal measurements on admission to the hospital and prior to delivery, but also to the measurements of the newborns. Consequently, it remains unclear when and how precisely these measurements were taken by the nurses. The extensive archive forms and the precise recording of all information and measurements suggest a standardized procedure. The visualizations of the distributions of waist circumferences suggest (Suppl. Fig. S4 in S1 File), given their shape, that these measurements were taken with sufficient precision.

#### Covariates.

Information related to the mother included date of birth, date of last menstrual period, age (years), height (cm), WC (cm), gravidity (categorized into 1, 2, > 2), civil status (categorized into “married” or “single”, the latter grouping “single”, “widowed” or “divorced” mothers), occupation and residential address at the time of delivery (categorized into “living inside Lausanne”: “yes”, “no”, or “unsure”). Neonatal variables included birth weight in grams, head circumference in cm, birth length in cm, placenta weight in grams, gestational age (GA) assessed at birth, neonatal sex, stillbirth (live-born or stillborn status immediately after delivery) and early neonatal mortality. Because almost all mother-child pairs stayed at the maternity at least 5 days after delivery, early neonatal mortality corresponds to mortality over that period. Maternal gravidity count included previous stillbirths and miscarriages. Preterm birth (PTB) was defined as GA less than 37 weeks based on the date of last menstruation, unless this derived GA was inconsistent with the GA assessed at birth: in that case we used GA assessed at birth. Qualitative information about the woman’s general health status was written in a dedicated section, from which we created the binary variables goitre, rickets, and a categorical variable describing maternal morphology (“thin”, “obese” or “neither”). A variable “infection” was created which took the value “yes” if flu, syphilis, gonorrhoea or tuberculosis were mentioned during pregnancy or at delivery, based on information written in the “general health status” section or in the one related to the course of the pregnancy. Maternal occupation was coded using the Historical International Standard of Classification of Occupations (HISCO) database [33]. HISCO codes were then grouped into 4 classes: 1 for non-manual occupations, higher managers/professionals, 2 for medium-skilled workers, farmers, and 3 for unskilled workers/farm workers, and missing when occupation was not reported. Seasonality was categorized based on birth month: March-May (spring), June-August (summer), September-November (autumn), and December-February (winter).

### 2.2. Dependent variables

Birth weight, head circumference, birth length and ponderal index ($\frac{\text{100}*\text{birth weight }(\text{g})}{\text{birth leng}{\text{th }(\text{cm})}^{\text{3}}}$) (PI) were used as continuous dependent variables. PTB, stillbirth, early neonatal mortality in the first 5 days of life, macrosomia (birth weight>95^th^ percentile which actually corresponds to 4,000g in our sample, a threshold also used in studies to define macrosomia [34]), microcephaly (head circumference sex-specific z-score below two standard deviations) and low birth weight (<2,500g) were used as binary dependent variables.

### 2.3. Independent variable

We first explored different independent variables. In linear univariable plots, we investigated the association between maternal height, WC or of the ratio between these two measures and the continuous dependent variables (birth weight, head circumference, birth length and PI). The univariable relationship between maternal and neonatal anthropometrics can be seen in Suppl. Fig. S1 in S1 File. Pearson’s correlation coefficients between these variables were also calculated (Suppl. Table S1 in S1 File). We chose to keep WC as the main independent variable in the rest of the analysis: it had the highest Pearson’s correlation coefficient for all neonatal anthropometrics variables, compared to the other maternal anthropometrics.

We further explored whether the relationship between maternal WC and neonatal size was linear by fitting univariable generalized additive models (GAMs) [35] with maternal size as the independent and neonatal size as the dependent variable (Suppl. Fig. S2 in S1 File). We see that at the two extremes of WC distribution (WC < 80 cm or >110 cm), neonatal size seems to reach a plateau. In between these extremes (waist circumference between 80 and 110 cm), neonatal size seems to be increasing non-linearly with waist circumference. Thus, WC was categorized into: < 80, 80–95, 95–110, and >110 cm. This categorizing allows taking into account the non-linear relationships, but also makes is easier to communicate our results, interpreting our outcomes by 15 cm increases in waist circumference.

Almost all mothers described as thin had a WC < 95 cm, and those described as obese had a WC > 95 cm (Suppl. Fig. S3 in S1 File). However, information on maternal morphology was qualitative (probably based on visual assessment) and not based on maternal body weight, which was not reported. We thus chose not to consider morphology in the rest of the analyses.

### 2.4. Exclusions

From the initial dataset (4,481 births between 1917 and 1921), we excluded births that took place at home (n = 30, 0.67%) and infants with birth weight < 500g or gestational age < 22 weeks (*n* = 3, 0.07%), which correspond to the current definition of miscarriage [36]. The final dataset for our study population thus consists of 4,448 births.

### 2.5. Statistical methods

Multiple imputation of missing data: The pattern of missing values in the variables of interest can be seen in Suppl. Fig. S4 in S1 File. Multiple imputation by chained equations (MICE) is a widely used and efficient method for imputing missing data [37]. We applied it primarily to handle missing WC values, but also to complete birth weight, head circumference, birth length, sex, maternal age and height variables. Some of these variables are also our dependent variables: it has been shown that using the dependent variable in imputation models leads to more valid results [38]. Since the fraction of missing values for waist circumference was 4.7%, we imputed 10 datasets each with 10 iterations. All continuous variables were imputed using predictive mean matching (“pmm” method) and the binary variable sex was imputed using logistic regression (“logreg” method).

Unadjusted differences in pregnancy outcomes depending on maternal WC were assessed with Wilcoxon rank sum test, Pearson’s Chi-squared test, and Fisher’s exact test.

Models: Generalized linear models (GLMs) were built, using a Gaussian family for the continuous dependent variable models and a logistic family with a log link for the binary dependent variable models. GLM models using imputed datasets were run with the function with.mids(). GLM results for each imputed dataset were pooled together using the function pool(), according to Rubin’s rule [39]. Univariable GLMs were built to model the relationship between WC and birth weight, birth length, head circumference, PI, LBW, PTB, stillbirth, early neonatal death in the first five days of life, microcephaly and macrosomia. WC was categorized as <80 cm, 80–95 cm, 95–110 cm or >110 cm in all models except for the PTB and microcephaly outcome models. For these models, since there were no cases of PTB or microcephaly among mothers with WC > 110 cm, only three categories were used: < 80 cm, 80–95 cm and >95 cm. Multivariable GLMs were built to model the relationship between WC and birth weight, birth length, head circumference, PI, LBW and PTB, adjusted for maternal age, civil status, gravidity, living in Lausanne, birth year, birth season, infection and infant sex. The other dependent variables were not modelled in multivariable models because of low number of events (stillbirth, neonatal death, microcephaly, macrosomia). For binary outcomes, marginal probabilities and marginally adjusted probabilities, respectively from the same univariable and multivariable GLMs than described above, were reported.

The question of whether gestational age should be adjusted for merits thorough attention. GA is an important determinant of neonatal size [40], and determines the time at which neonatal size is measured (at delivery). But gestational age can itself depend on maternal waist circumference. Gestational age is thus a confounder of the association between waist circumference and birth weight (Suppl Figure 5A in S1 File). Adjusting for gestational age would thus seem reasonable, closing the biasing path (Suppl Figure 5B in S1 File). However, various maternal factors, such as maternal weight or weight gain during pregnancy, can make both gestational age and birth weight vary [41,42]. If maternal weight is known and can be adjusted for, no biasing path is open (Suppl Figure 5C in S1 File). However, if maternal weight cannot be measured and gestational age is adjusted for, a colliding path opens (Suppl Figure 5D in S1 File). Therefore, adjusting or stratifying for gestational age (the collider) in the regression models may introduce collider bias [43–45]. This will be the case for any variable which makes both gestational age and birth weight vary and that cannot be adjusted for. In this sense, it is advisable to not adjust for gestational age (Suppl Figure 5E in S1 File).

Sensitivity analyses: All analyses were also run on the complete-case dataset (i.e., pre-imputation and excluding the missing cases). In addition, all analyses were repeated with births from primi gravida women only, so that each woman only appears once. The effect of waist circumference on all outcomes was also modelled using a spline in multivariable analyses, with two knots positioned at 80 cm and 110 cm, on the imputed datasets.

All analyses were performed using R version 4.3.2 [46]. Packages used were mice[47] for the imputation, mgcv [35] for the GAM models, and emmeans for the marginal probabilities.

## 3. Results

Between 1917 and 1921, *n* = 4,448 singleton births took place at the maternity hospital. The maternal and child characteristics are described in **Table 1**. After imputation, the mothers were on average 28.4 years old (same than before imputation), 156.3 cm tall (156.2 cm before imputation), mostly married (87%), belonged to HISCO class 2 (87%), and did not live in the city of Lausanne (63%). Among the births, the number of primi gravida women was similar to the women who already had two pregnancies (40% vs. 39%). At birth, 6% of the women had a goiter, 4% had rickets and 10% had an infection, according to the physicians at the time. The mean WC of the mothers shortly before birth was 94.9 cm (SD 7.5), similar to the one before imputation (95.0 cm, SD 7.5). In the imputed as well as in the complete-case dataset, only 1% of women (n = 66) had a WC below 80 cm and 3% (n = 124) had a WC over 110 cm. Slightly more newborn infants were boys (52%, both before and after imputation), and 9% of the infants were born before term (<37 weeks). The infants weighed an average of 3,177.7 grams (3,178.0 before imputation) and had a head circumference of 34.3 cm (34.4 before imputation). Around 4% of the infants were stillborn, and around 2% died in the first five days after birth. Low birthweight affected 9% of the infants, and macrosomia 5% (same figures before and after imputation). Maternal and infant characteristics before and after imputation were therefore virtually the same (**Table 1**).

**Table 1:** Descriptive table of the dataset before and after imputation ^1^Mean (SD); n (%).

Maternal characteristics, *n* = ,448				Neonatal characteristics, *n* = 4,448		
Variable		Before imputation^1^	After imputation^1^	Variable	Before imputation^1^	After imputation^1^
maternal age (years)		28.4 (6.2)	28.4 (6.2)	birth weight (g)	3,178.0 (561.6)	3,177.7 (561.8)
missing		8		missing	4	
height (cm)		156.2 (6.6)	156.3 (6.6)	head circumference (cm)	34.4 (1.8)	34.3 (1.9)
missing		182		missing	71	
waist circumference (cm)		95.0 (7.5)	94.9 (7.5)	birth length (cm)	49.1 (2.7)	49.1 (2.7)
missing		208		missing	19	
civil status				sex		
married		3,871 (87%)	3,871 (87%)	male	2,286 (52%)	2,298 (52%)
single or missing		577 (13%)	577 (13%)	female	2,146 (48%)	2,150 (48%)
gravidity				missing	16	
1		1,777 (40%)	1,777 (40%)	gestational age (weeks)		
2		958 (22%)	958 (22%)	<33	114 (3%)	114 (3%)
>2		1,713 (39%)	1,713 (39%)	33-37	296 (7%)	296 (7%)
living in Lausanne				37-41	3,787 (85%)	3,787 (85%)
no		2,781 (63%)	2,781 (63%)	>41	251 (6%)	251 (6%)
unsure		64 (1%)	64 (1%)	stillbirth	164 (4%)	164 (4%)
yes		1,603 (36%)	1,603 (36%)	neonatal death d1-5	97 (2%)	97 (2%)
goitre		261 (6%)	261 (6%)	missing	4	4
rickets		192 (4%)	192 (4%)	preterm birth (<37 weeks)	410 (9%)	410 (9%)
infection		442 (10%)	442 (10%)	low birth weight (<2,500g)	389 (9%)	390 (9%)
HISCO class				missing	4	
1		265 (6%)	265 (6%)	macrosomia (>95th percentile)	208 (5%)	208 (5%)
2		3,852 (87%)	3,852 (87%)	missing	4	
3		84 (2%)	84 (2%)	ponderal index	2.67 (0.27)	2.67 (0.27)
missing		247 (6%)	247 (6%)	missing	22	
waist circumference categories (cm)				microcephaly (Z-score < −2 sd)	124 (3%)	145 (3%)
<80		62 (1%)	66 (1%)	missing	71	
80-95		2,276 (54%)	2,394 (54%)			
95-110		1,785 (42%)	1,864 (42%)			
>110		117 (3%)	124 (3%)			
missing		208				

Because the association of all neonatal anthropometrics were stronger with WC than with maternal height or with the ratio WC/height (Fig. S1 and S2, Table S1 in S1 File), WC was chosen as the main independent variable. The maternal WC was categorised for further modelling, based on visual assessment that the relationship between WC and neonatal anthropometrics was non-linear between 80 and 110 cm, and reached a plateau below and above these values (Fig. S2 in S1 File); an additional cut-off was chosen as the centre of 80–100 cm, in order to have equal-sized categories: < 80, 80–95, 95–110, > 110 cm (Fig. S2 in S1 File). The association between categorized WC and various birth outcomes is described in **Table 2**. With higher WC category, all infant anthropometric measurements were higher, while the rates of premature births, low birth weight and microcephaly were lower. The frequency of stillbirth was highest in the lowest and highest WC categories (u-shaped association), but there was no clear evidence of an association. When using complete case analysis only, the same observations can be made (Table S2 in S1 File).

**Table 2:** Neonatal outcomes depending on maternal waist circumference, after MICE.

Variable	Waist circumference (cm)						
	<80, N = 66		80-95, N = 2,394 *(reference)*	95-110, N = 1,864		>110, N = 124	
	value^*1*^	p-value^*2*^	value^*1*^	value^*1*^	p-value^*2*^	value^*1*^	p-value^*2*^
birth weight (g)	2,409.2 (728.8)	<0.001	3,018.7 (536.6)	3,377.6 (488.3)	<0.001	3,649.1 (527.3)	<0.001
head circumference (cm)	31.9 (3.1)	<0.001	33.9 (1.8)	34.9 (1.7)	<0.001	35.6 (1.5)	<0.001
birth length (cm)	45.4 (4.3)	<0.001	48.4 (2.8)	49.9 (2.1)	<0.001	51.0 (2.0)	<0.001
sex		>0.9			<0.001		0.031
male	32 (48%)		1,172 (49%)	1,021 (55%)		73 (59%)	
female	34 (52%)		1,222 (51%)	843 (45%)		51 (41%)	
gestational age (weeks)		<0.001			<0.001		<0.001
<33	12 (18%)		81 (3%)	21 (1%)		0 (0%)	
33-37	20 (30%)		220 (9%)	56 (3%)		0 (0%)	
37-41	33 (50%)		2,024 (85%)	1,629 (87%)		101 (81%)	
>41	1 (2%)		69 (3%)	158 (8%)		23 (19%)	
stillbirth	5 (8%)	0.091	85 (4%)	66 (4%)	>0.9	8 (6%)	0.13
neonatal death d1-5	6 (9%)	0.006	57 (2%)	31 (2%)	0.10	3 (2%)	>0.9
missing	0		3	1		0	
preterm birth (<37 weeks)	32 (48%)	<0.001	301 (13%)	77 (4%)	<0.001	0 (0%)	<0.001
low birth weight (<2,500g)	31 (47%)	<0.001	294 (12%)	62 (3%)	<0.001	3 (2%)	<0.001
macrosomia (>95th percentile)	1 (2%)	0.6	32 (1%)	147 (8%)	<0.001	28 (23%)	<0.001
ponderal index	2.49 (0.37)	<0.001	2.63 (0.26)	2.71 (0.26)	<0.001	2.75 (0.29)	<0.001
microcephaly (Z-score < −2 sd)	17 (26%)	<0.001	102 (4%)	26 (1%)	<0.001	0 (0%)	0.019

^*1*^Mean (SD); n (%). ^*2*^ Wilcoxon rank sum test; Pearson’s Chi-squared test; Fisher’s exact test (comparing each waist circumference category with the category 80-95 cm).

**Fig 1:**
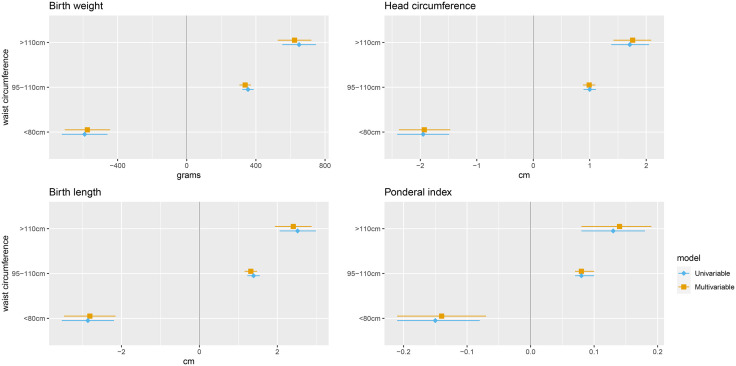
Relationship between waist circumference and infant size (estimates and their 95% CI). These are pooled results from univariable and multivariable GLMs ran on imputed datasets. Multivariable GLMs are adjusted for maternal age, civil status, gravidity, living in Lausanne, birth year, birth season, infection and infant sex. The reference category for waist circumference is 80-95 cm.

**Fig 2:**
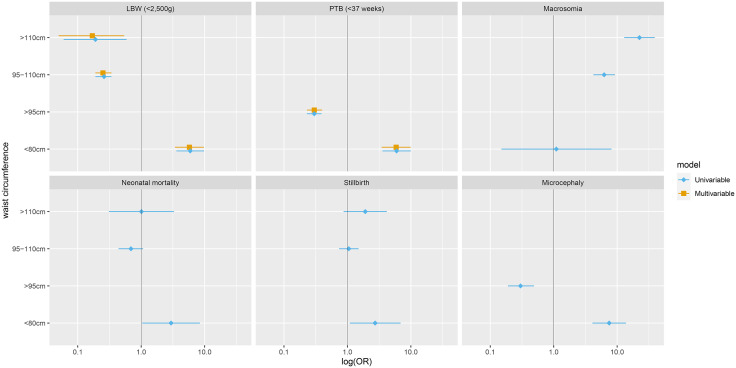
Relationship between waist circumference and binary pregnancy outcomes (OR and their 95% CI). These are pooled results from univariable and multivariable GLMs ran on imputed datasets. Multivariable GLMs are adjusted for maternal age, civil status, gravidity, living in Lausanne, birth year, birth season, infection and infant sex. The reference category for waist circumference is 80-95 cm. Due to the low number of events, less waist circumference categories are used for the preterm birth and microcephaly outcomes.

The relationship between WC and neonatal outcomes were further investigated in univariable and multivariable generalized linear models (GLMs) (Tables 3 and 4, Fig 1 and 2, full models for multivariable GLMs and complete-case analyses are displayed in Suppl. Tables S3-10 in S1 File). The univariable and multivariable models show very similar results for the continuous dependent variables measures. For example, regarding birth weight in the univariable model, a WC < 80 cm was associated with a birth weight lower by −590.9 grams (95%CI −723.1 to −458.6) compared to the reference category of 80–95 cm, while a WC > 110 cm was associated with a birth weight higher by +649.8 grams (+552.5 to + 747.2). The models for head circumference, birth length and PI similarly show lower (higher) neonatal anthropometrics in the case of WC < 80 cm (>110 cm).

**Table 3:** Relationship between waist circumference and infant size.

			Univariable 95% CI				Multivariable 95% CI			
	Variable	Category	beta	lci	uci	p_value	beta	lci	uci	p_value
Birth weight (g)	(Intercept)		3020.35	2999.09	3041.61	<0.0001	3248.68	3153.66	3343.70	<0.0001
	waist circumference (ref: 80–95 cm)	<80 cm	−590.85	−723.08	−458.62	<0.0001	−575.17	−706.18	−444.17	<0.0001
		95-110 cm	354.19	321.58	386.79	<0.0001	337.85	304.66	371.04	<0.0001
		>110 cm	649.83	552.49	747.17	<0.0001	623.42	526.14	720.70	<0.0001
	(Intercept)		33.90	33.83	33.97	<0.0001	34.85	34.52	35.17	<0.0001
Head circumference (cm)	waist circumference (ref: 80–95 cm)	<80 cm	−1.95	−2.41	−1.49	<0.0001	−1.93	−2.38	−1.47	<0.0001
		95-110 cm	1.00	0.89	1.11	<0.0001	0.99	0.88	1.10	<0.0001
		>110 cm	1.71	1.38	2.05	<0.0001	1.76	1.42	2.09	<0.0001
Birth length (cm)	(Intercept)		48.46	48.35	48.56	<0.0001	49.36	48.90	49.82	<0.0001
	waist circumference (ref: 80–95 cm)	<80 cm	−2.86	−3.53	−2.19	<0.0001	−2.81	−3.47	−2.15	<0.0001
		95-110 cm	1.39	1.23	1.55	<0.0001	1.32	1.16	1.48	<0.0001
		>110 cm	2.52	2.06	2.99	<0.0001	2.41	1.94	2.88	<0.0001
Ponderal index	(Intercept)		2.63	2.62	2.64	<0.0001	2.71	2.66	2.76	<0.0001
	waist circumference (ref: 80–95 cm)	<80 cm	−0.15	−0.21	−0.08	<0.001	−0.14	−0.21	−0.07	<0.001
		95-110 cm	0.08	0.07	0.10	<0.0001	0.08	0.07	0.10	<0.0001
		>110 cm	0.13	0.08	0.18	<0.0001	0.14	0.08	0.19	<0.0001

These are pooled results from univariable and multivariable GLMs ran on imputed datasets. Multivariable GLMs are adjusted for maternal age, civil status, gravidity, living in Lausanne, birth year, birth season, infection and infant sex.

**Table 4:** Relationship between waist circumference and binary pregnancy outcomes. These are pooled results from univariable and multivariable logistic GLMs ran on imputed datasets. Multivariable GLMs are adjusted for maternal age, civil status, gravidity, living in Lausanne, birth year, birth season, infection and infant sex. Due to the low number of events, less waist circumference categories are used for the preterm birth and microcephaly outcomes. Marginal probabilities are reported in Suppl. Table S11 in S1 File.

			Univariable 95% CI				Multivariable 95% CI			
Outcome variable	Variable	Category	OR	lci	uci	p_value	OR	lci	uci	p_value
Low birth weight (<2,500g)	(Intercept)		0.14	0.12	0.16	<0.0001	0.03	0.02	0.06	<0.0001
	waist circumference (ref: 80–95 cm)	<80 cm	5.93	3.59	9.79	<0.0001	5.76	3.42	9.71	<0.0001
		95-110 cm	0.26	0.19	0.34	<0.0001	0.25	0.19	0.34	<0.0001
		>110 cm	0.19	0.06	0.59	<0.01	0.17	0.05	0.54	<0.01
	(Intercept)		0.14	0.13	0.16	<0.0001	0.08	0.04	0.16	<0.0001
Preterm birth (<37 weeks)	waist circumference (ref: 80–95 cm)	<80 cm	5.96	3.57	9.95	<0.0001	5.86	3.46	9.93	<0.0001
		>95 cm	0.30	0.23	0.39	<0.0001	0.30	0.23	0.40	<0.0001
Macrosomia (>95^th^ percentile)	(Intercept)		0.01	0.01	0.02	<0.0001	–			
	waist circumference (ref: 80–95 cm)	<80 cm	1.10	0.15	8.15	0.93				
		>95 cm	6.24	4.22	9.24	<0.0001				
		>110 cm	22.48	12.92	39.13	<0.0001				
Microcephaly (Z-score < 2sd)	(Intercept)		0.04	0.04	0.05	<0.0001	–			
	waist circumference (ref: 80–95 cm)	<80 cm	7.49	4.08	13.75	<0.0001				
		>95 cm	0.30	0.19	0.49	<0.0001				
Stillbirth	(Intercept)		0.04	0.03	0.04	<0.0001	–			
	waist circumference (ref: 80–95 cm)	<80 cm	2.73	1.09	6.87	0.03				
		95-110 cm	1.05	0.74	1.50	0.79				
		>110 cm	1.91	0.87	4.18	0.11				
Neonatal mortality day1–5	(Intercept)		0.02	0.02	0.03	<0.0001	–			
	waist circumference (ref: 80–95 cm)	<80 cm	2.96	1.04	8.42	0.04				
		95-110 cm	0.69	0.44	1.07	0.10				
		>110 cm	1.01	0.31	3.29	0.98				

The models for the binary pregnancy outcomes are shown in Table 4 and Fig 2. In univariable models, a maternal WC < 80 cm was associated with a greater probability of LBW (OR 5.93, 95%CI 3.59 to 9.79). In the complete dataset, the marginalized probability of LBW is 43.6% [95 CI 31.8 to 56.0%], 11.2% [95%CI 10.0 to 12.6%], 3.4% [95%CI 2.6 to 4.3%] and 2.6% [0.8 to 7.7%] among mothers with a waist circumference <80, 80–95, 95–110, and >110 cm, respectively (Suppl. Table S11 in S1 File). A maternal WC < 80 cm was also associated with higher odds of PTB (OR 5.96, 95%CI 3.57 to 9.95), microcephaly (OR 7.49, 95%CI 4.08 to 13.75), stillbirth (OR 2.73, 95%CI 1.09 to 6.87) and neonatal mortality (OR 2.96, 95%CI 1.04 to 8.42), as compared to the reference category. In contrast, a WC of >110 cm or >95 cm, depending on the model, was associated with more favorable pregnancy outcomes, except for the probability of macrosomia which was higher: OR= 22.48 (95%CI 12.92 to 39.13) in the largest WC category (>110 cm).

The sensitivity analysis with only first pregnancies and the models with the complete cases before imputation led to the same conclusion (Tables S12-S13 and Tables S3-S10 in S1 File). When WC was modelled using a spline, results were generally consistent with the main analysis using categorized WC. However, when WC was higher than 120 cm, detrimental effects started to be noticed, with increasing risks of LBW, PTB, macrosomia, microcephaly, neonatal mortality and stillbirth (Suppl. Figures S6-7 and Tables S14-15 in S1 File); confidence intervals were however large, and it should be noted that only 14 women had such a WC > 120 cm. In addition, birth weight was higher for mothers with a WC = 110 (+405g [95%CI 365 to 446g]), than for mothers with a WC = 120 (371g [95%C 238 to 503g]), cm compared to the reference WC of 95 cm (median of the sample).

## 4. Discussion

In this study, we took advantage of the fact that over 100 years ago, and thus long before the introduction of today’s prenatal diagnostic methods in Switzerland, a large number of maternal and infant characteristics were recorded, measured and registered at births in Lausanne maternity hospital. In a dataset of over 4,400 mother-child pairs in Lausanne, we were able to show that the maternal WC, measured shortly before delivery, was associated with the size of the infant at birth and the occurrence of adverse pregnancy outcomes. In mothers with a comparatively small pre-delivery WC, birth weight, length, head circumference and the PI of their newborns were significantly lower, and the probability of LBW, PTB, neonatal mortality, stillbirth and microcephaly was higher. In contrast, in mothers with a particularly large pre-delivery abdominal girth, the newborns were larger, and the likelihood of macrosomia was higher. This indicates that in this historical setting pre-delivery maternal WC shortly before birth was associated with certain subsequent birth outcomes.

To the best of our knowledge, there is no other modern study that has examined comparable historical data with a similar research question. Therefore, we cannot compare our results with other historical epidemiological studies. Although a comparison with modern data would be possible and interesting in follow-up studies, it should be noted that the context of life in Switzerland has fundamentally changed over the last 100 years, as mentioned in the introduction. While today’s studies focus on obesity as a major risk factor, this issue was much less relevant 100 years ago, when undernutrition was a more important and frequent public health problem than overnutrition [21].

Furthermore, the comparison with modern data and studies would also be made more complex by the lack of longitudinal information on changes in maternal and fetal body size during pregnancy in the historical data, which would have allowed a more precise examination of maternal weight gain during pregnancy, for example. Women with a higher pre-pregnancy WHR have been shown in modern-times studies to face an higher likelihood of giving birth to macrosomic infants [8]. Unfortunately, pre-pregnancy WC is virtually always absent of historical datasets. In other recent studies, it has been shown that maternal undernutrition during pregnancy results in fetal growth restriction with increased risk of stillbirth, neonatal mortality and morbidity, inhibited growth and cognitive development, and chronic diseases later in life [13,17,18]. It is thus possible that the detrimental neonatal effects associated with a particularly low or high WC that we document in this study have led to poor children health.

In terms of interpretability, it should be mentioned that WC measured shortly before delivery is not the best indicator of fetal size, because it is influenced by several contributing factors such as maternal body habitus, uterine volume, amniotic fluid, and gestational age [48]. The observed associations are therefore complex to interpret biologically in a specific or causal way, and this parameter may not directly reflect more than fetal growth restriction or maternal nutritional status. In addition, reverse causality may also be a limitation: a larger fetal weight will indeed directly increase waist circumference.

As justified in the *Methods* section, we chose not to adjust the analyses for GA, to avoid collider bias. However, since GA is a strong determinant of fetal growth and health, being born earlier results in a lower birth weight and size. Thus, our findings of lower infant weight and size when maternal WC is low may be – at least partly – mediated by shorter gestation. On the other hand, the finding of higher macrosomia risk for high maternal WC may be – at least partly- mediated by longer gestation. As an important constraint, the historical birth records of Lausanne’s maternity hospital do not adequately reflect certain maternal factors that can have a strong influence on maternal and fetal health during pregnancy and there may be residual confounders such as maternal BMI, diabetes, metabolic status, and information on smoking and nutrition which limit the ability to draw causal conclusions. This also applies to so-called intergenerational and/or lifestyle factors: Birthweight is affected to a great extent by the mother’s own fetal growth and her diet from birth to pregnancy, and thus, her body composition at conception [49,50]. Once pregnant, the mother’s nutrition and diet, lifestyle (e.g., stress, physical workload, alcohol, or drug abuse) and other exposures (e.g., infectious diseases), or complications such as hypertension can affect fetal growth and development, as well as the duration of pregnancy [13].

Our study has several further limitations: Firstly, a sample selection bias may affect our dataset. Although two-thirds of all births in the city of Lausanne occurred at the maternity hospital at the beginning of the 1920s [29], we have no information regarding the remaining home births. It is possible that difficult and higher-risk births were more common in hospital births than in the home births, and that this bias also affected the association WC and pregnancy outcomes. Secondly, despite intensive archive research, hospital guidelines or documentation on measurement protocols for maternal WC and other anthropometric indicators could not be found. Thus, it must remain unclear how this measurement was exactly performed, at which anatomical level, and under what clinical circumstances. Without a clearly documented measurement protocol, the variable may be affected by measurement and interobserver variability and may not correspond to a reproducible anthropometric construct in the modern sense. Thirdly, this study uses data from one single hospital at a specific point in time in a historical Swiss population where undernutrition was common, which is different from today’s population. We cannot say whether the results from Lausanne can be generalized to other hospitals, the population as a whole, or earlier or later points in time. To test generalizability and the relevance of our findings to modern obstetrics, we would need further comparable data sets from more hospitals and also modern times.

## 5. Conclusion

Our study suggests that pre-delivery maternal WC shortly before birth was associated with certain subsequent birth outcomes in a historical setting in Switzerland around 1920. This relates to a lifestyle context in which certain risk factors, such as obesity, were not as prevalent as they are today.

## Supporting information

S1 File Supplementary Material containing all Supplementary Tables S1-15 and Figures S1-7. (DOCX)

## References

[1] Achievements in public health, 1900-1999: Healthier mothers and babies. Morb Mortal Wkly Rep. 1999;48:30333.

[2] Ward J. Global, regional, and national mortality among young people aged 10–24 years, 1950–2019: a systematic analysis for the Global Burden of Disease Study 2019. The Lancet. 2021;:1593–618. doi: 10.1016/S0140-6736(21)01546-4 PMC8576274 34755628

[3] Brown JE, Potter JD, Jacobs D. Maternal Waist to Hip Ratio as a Predictor of Newborn Size. Epidemiology Resources Inc. 1996;7. 10.1097/00001648-199601000-00011 8664403

[4] Boucher T, Farmer L, Moretti M, Lakhi NA. Maternal anthropometric measurements and correlation to maternal and fetal outcomes in late pregnancy. Women’s health. 2022. doi: 10.1177/17455065221076737 PMC8814965 35107042

[5] Hancerliogullari N, Kansu-celik H, Kaplan ZAO, Ozgu-erdinc AS, Engin-ustun Y. Correlation of maternal neck/waist circumferences and fetal macrosomia in low-risk Turkish pregnant women, a preliminary study. Fetal Pediatr Pathol. 2021;40:181–8. 31603015 10.1080/15513815.2019.1675831

[6] Okereke CE, Anyaehie UB, Dim CC, Iyare EE, Nwagha UI. Evaluation of some anthropometric indices for the diagnosis of obesity in pregnancy in Nigeria: a cross-sectional study. Afr Health Sci. 2013;13(4):1034–40. doi: 10.4314/ahs.v13i4.25 24940329 PMC4056469

[7] Jokelainen M, Stach-Lempinen B, Teramo K, Nenonen A, Kautiainen H, Klemetti MM. Large maternal waist circumference in relation to height is associated with high glucose concentrations in an early-pregnancy oral glucose tolerance test: A population-based study. Acta Obstet Gynecol Scand. 2023;102(4):496–505. doi: 10.1111/aogs.14528 36799298 PMC10008291

[8] Salem W, Adler AI, Lee C, Smith GCS. Maternal waist to hip ratio is a risk factor for macrosomia. BJOG. 2012;119(3):291–7. doi: 10.1111/j.1471-0528.2011.03167.x 22004312

[9] Haag N, Le Vu M, Jaeggi AV, Baud D, Desseauve D, Haeusler M, et al. Association between a mismatch of maternal/neonatal body size and obstetrical interventions in Switzerland in the 1920s: a cross-sectional study. Swiss Med Wkly. 2025;155:4546. doi: 10.57187/s.4546 41364818

[10] Bocquenet G. A fair chance for every child. 2016.

[11] Countdown to 2030 Collaboration. Countdown to 2030: tracking progress towards universal coverage for reproductive, maternal, newborn, and child health. Lancet. 2018;391(10129):1538–48. doi: 10.1016/S0140-6736(18)30104-1 29395268

[12] Bocquenet G. A fair chance for every child. 2016.

[13] Bhowmik B, Siddique T, Majumder A, Mdala I, Hossain IA, Hassan Z, et al. Maternal BMI and nutritional status in early pregnancy and its impact on neonatal outcomes at birth in Bangladesh. BMC Pregnancy Childbirth. 2019;19(1):413. doi: 10.1186/s12884-019-2571-5 31711436 PMC6849244

[14] Wardlaw T, Blanc A, Zupan J, Åhman E. United Nations Childresn’s Fund and World Health Organization Low Birthweight: Country, Regional and Global Estimates. New York: UNICEF. 2004. doi: 10.2307/2800038

[15] Satpathy HK, Fleming A, Frey D, Barsoom M, Satpathy C, Khandalavala J. Maternal obesity and pregnancy. Postgrad Med. 2008;120(3):E01–9. doi: 10.3810/pgm.2008.09.1920 18824817

[16] Cisneiros RM, Dutra LP, Silveira FJC, Souza AR, Marques M, Amorim MM, et al. Visceral adiposity in the first half of pregnancy predicts newborn weight among adolescent mothers. J Obstet Gynaecol Can. 2013;35(8):704–9. doi: 10.1016/S1701-2163(15)30860-4 24007705

[17] Black RE, Allen LH, Bhutta ZA, Caulfield LE, de Onis M, Ezzati M, et al. Maternal and child undernutrition: global and regional exposures and health consequences. Lancet. 2008;371(9608):243–60. doi: 10.1016/S0140-6736(07)61690-0 18207566

[18] Wardlaw T, Blanc A, Zupan J, Åhman E. United Nations Childresn’s Fund World Health Organization Low Birthweight: Country, Regional and Global Estimates. New York: UNICEF. 2004. doi: 10.2307/2800038

[19] NCD Risk Factor Collaboration (NCD-RisC). Worldwide trends in body-mass index, underweight, overweight, and obesity from 1975 to 2016: a pooled analysis of 2416 population-based measurement studies in 128·9 million children, adolescents, and adults. Lancet. 2017;390(10113):2627–42. doi: 10.1016/S0140-6736(17)32129-3 29029897 PMC5735219

[20] Matthes KL, Hartmann C, Siegrist M, Burnier M, Bochud M, Zwahlen M, et al. A quantitative synthesis study on body mass index and associated factors among adult men and women in Switzerland. J Nutr Sci. 2022;11:e65. doi: 10.1017/jns.2022.66 35992574 PMC9379928

[21] Staub K, Bender N, Floris J, Pfister C, Rühli FJ. From Undernutrition to Overnutrition: The Evolution of Overweight and Obesity among Young Men in Switzerland since the 19th Century. Obes Facts. 2016;9(4):259–72. doi: 10.1159/000446966 27544200 PMC5644905

[22] Floris J, Höpflinger F, Stohr C, Studer R, Staub K. Wealthier – older – taller: measuring the standard of living in Switzerland since the 19th century. SZG/RSH/RSS. 2019;2:207–32.

[23] Staub K. Der vermessene menschliche Körper als Spiegel der Ernährungs- und Gesundheitsverhältnisse am Ende des Ersten Weltkrieges. In: Schwabe, editor. «Woche für Woche neue Preisaufschläge». Nahrungsmittel-, Energie- und Ressourcenkonflikte in der Schweiz des Ersten Weltkrieges. Basel. 2016:285–305.

[24] Ammon CE. The 1918 Spanish flu epidemic in Geneva, Switzerland. Int Congr Ser. 2001;1219.

[25] Staub K, Floris J, Woitek U, Rühli F. From left-skewness to symmetry: how body-height distribution among Swiss conscripts has changed shape since the late 19th century. Ann Hum Biol. 2015;42(3):260–7. doi: 10.3109/03014460.2014.942366 25154618

[26] Koepke N, Floris J, Pfister C, Rühli FJ, Staub K. Ladies first: Female and male adult height in Switzerland, 1770-1930. Econ Hum Biol. 2018;29:76–87. doi: 10.1016/j.ehb.2018.02.002 29486413

[27] Floris J, Höpflinger F, Stohr C, Studer R, Staub K. Wealthier – older – taller: Measuring the standard of living in Switzerland since the 19th century. Schweizerische Zeitschrift für Geschichte. 2019. doi: 10.24894/2296-6013.00037

[28] Floris J., Staub K. Water, sanitation and mortality in Swiss towns in the context of urban renewal in the late nineteenth century. History of the Family. 2019;24. doi: 10.1080/1081602X.2019.1598460

[29] Registre de l’Etat Civil de Lausanne. Etat civil de Lausanne. 1920.

[30] Le Vu M, Cortina-Borja M, Wells JCK. Evaluating the Concept of Brain Sparing in a High Income Setting, Using Historical Records of Maternal Influenza or Syphilis Infection. Am J Hum Biol. 2024;36(11):e24143. doi: 10.1002/ajhb.24143 39180148

[31] Le Vu M, Matthes KL, Schneider EB, Moerlen A, Hösli I, Baud D, et al. Maternal Influenza-Like Illness and Neonatal Health During the 1918 Influenza Pandemic in a Swiss City. Ann Intern Med. 2025;178(11):1632–41. doi: 10.7326/ANNALS-24-03796 41052451

[32] Maternité de Lausanne. Obstétrique, K VIII e 227-255, 1917-1921. Accessed at https://davel.vd.ch/detail.aspx?ID=978319 on 7 July 2026.

[33] van Leeuwen MHD, Maas I, Miles A. Historical International Standard Classification of Occupations. Louvain: Leuven University Press. 2004.

[34] Damhuis SE, Ganzevoort W, Gordijn SJ. Abnormal fetal growth. Obstet Gynecol Clin North Am. 2021;48:267–79. 33972065 10.1016/j.ogc.2021.02.002

[35] Wood SN. Generalized Additive Models: An Introduction with R. 2017. doi: 10.1201/9781315370279

[36] Tavares Da Silva F, Gonik B, McMillan M, Keech C, Dellicour S, Bhange S, et al. Stillbirth: Case definition and guidelines for data collection, analysis, and presentation of maternal immunization safety data. Vaccine. 2016;34(49):6057–68. doi: 10.1016/j.vaccine.2016.03.044 27431422 PMC5139804

[37] van Buuren S. Flexible imputation of missing data. 2012.

[38] Moons KGM, Donders RART, Stijnen T, Harrell FE Jr. Using the outcome for imputation of missing predictor values was preferred. J Clin Epidemiol. 2006;59(10):1092–101. doi: 10.1016/j.jclinepi.2006.01.009 16980150

[39] Rubin DB. Inference and missing data. Biometrika. 1976;63(3):581.

[40] Wilcox AJ, Skjaerven R. Birth weight and perinatal mortality: the effect of gestational age. Am J Public Health. 1992;82(3):378–82. doi: 10.2105/ajph.82.3.378 1536353 PMC1694365

[41] Yu Z, Han S, Zhu J, Sun X, Ji C, Guo X. Pre-pregnancy body mass index in relation to infant birth weight and offspring overweight/obesity: a systematic review and meta-analysis. PLoS One. 2013;8(4):e61627. doi: 10.1371/journal.pone.0061627 23613888 PMC3628788

[42] Vats H, Saxena R, Sachdeva MP, Walia GK, Gupta V. Impact of maternal pre-pregnancy body mass index on maternal, fetal and neonatal adverse outcomes in the worldwide populations: A systematic review and meta-analysis. Obes Res Clin Pract. 2021;15(6):536–45. doi: 10.1016/j.orcp.2021.10.005 34782256

[43] Hernán MA, Hernández-Díaz S, Werler MM, Mitchell AA. Causal knowledge as a prerequisite for confounding evaluation: an application to birth defects epidemiology. Am J Epidemiol. 2002;155(2):176–84. doi: 10.1093/aje/155.2.176 11790682

[44] Skrivankova VW, et al. Sociodemographic and regional differences in neonatal and infant mortality in Switzerland: The Swiss National Cohort. 2023. doi: 10.1101/2023.09.19.23295765 39835837

[45] Delbaere I, Vansteelandt S, De Bacquer D, Verstraelen H, Gerris J, De Sutter P, et al. Should we adjust for gestational age when analysing birth weights? The use of z-scores revisited. Hum Reprod. 2007;22(8):2080–3. doi: 10.1093/humrep/dem151 17588952

[46] R Core Team. R: A Language and Environment for Statistical Computing. 2025. https://www.R-project.org/

[47] Van Buuren S, Groothuis-Oudshoorn K. Mice: Multivariate imputation by chained equations in R. Journal of Statistical Software. 2011;45.

[48] Dare FO, Ademowore AS, Ifaturoti OO, Nganwuchu A. The value of symphysio-fundal height/abdominal girth measurements in predicting fetal weight. Int J Gynaecol Obstet. 1990;31(3):243–8. doi: 10.1016/0020-7292(90)91018-l 1969365

[49] Wells JCK. Maternal capital and the metabolic ghetto: An evolutionary perspective on the transgenerational basis of health inequalities. Am J Hum Biol. 2010;22(1):1–17. doi: 10.1002/ajhb.20994 19844897

[50] Wells JCK, Stock JT. Re-examining heritability: genetics, life history and plasticity. Trends Endocrinol Metab. 2011;22(10):421–8. doi: 10.1016/j.tem.2011.05.006 21757369

