# Supplementary material for: Association between pre-birth waist circumference and neonatal size and health in mother-newborn pairs in Lausanne, 1917–1921
Source: PLoS One. 2026 Jul 13;21(7):e0352179. doi: 10.1371/journal.pone.0352179 (PMC13362130; doi:10.1371/journal.pone.0352179)
Supplement: S1 File — (DOCX) [file pone.0352179.s001.docx]

**Supplementary Material**

**Figure S1**: Univariable relationship between maternal anthropometrics (waist circumference, height or ratio waist circumference/height), and neonatal anthropometrics (birth weight, length, head circumference or ponderal index).


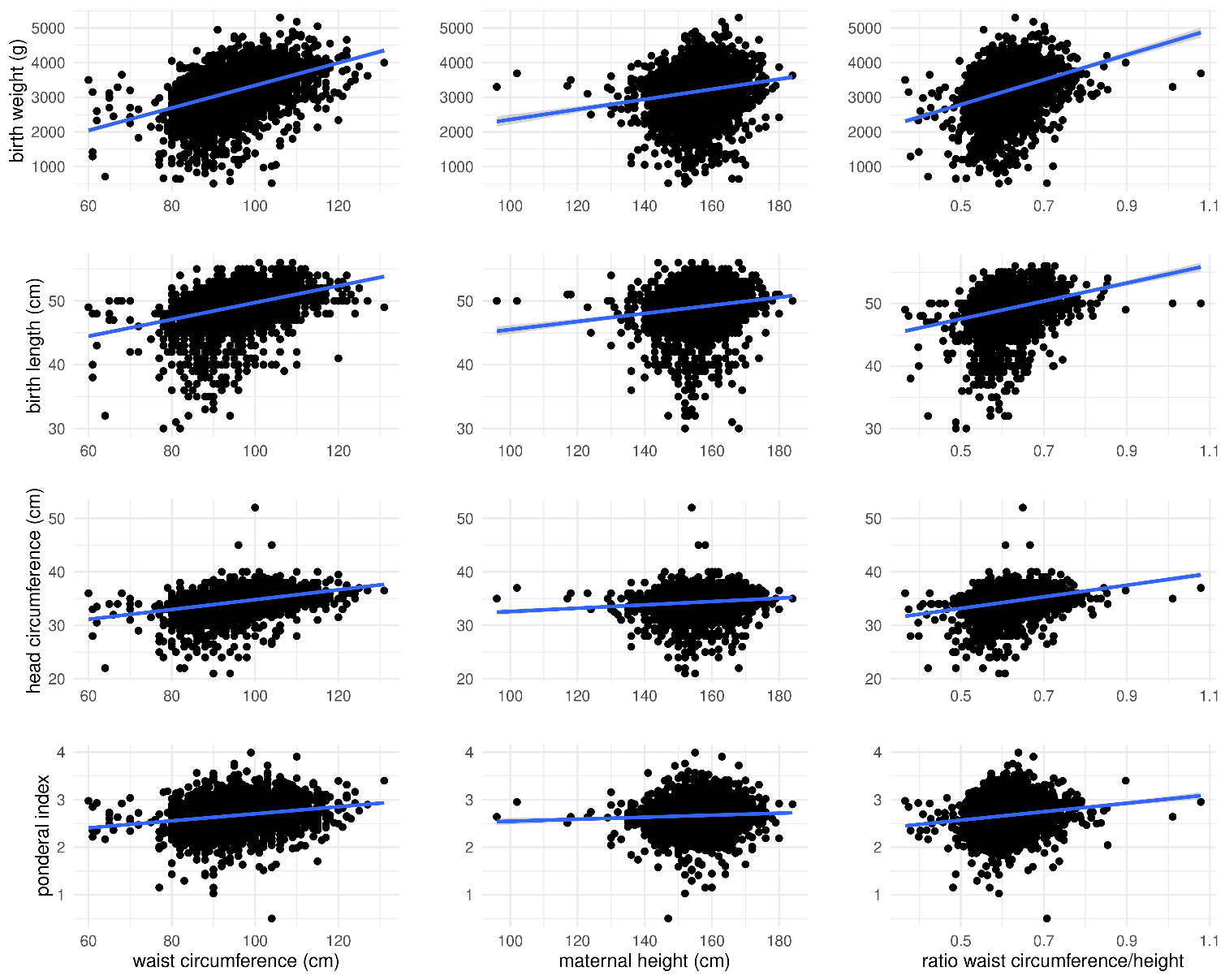


**Table S1**: Pearson’s correlation coefficient between maternal and neonatal anthropometric measures

|  |  | Neonatal anthropometric measures | | | |
| --- | --- | --- | --- | --- | --- |
|  |  | birth weight | birth length | head circumference | ponderal index |
| maternal anthropometric measures | waist circumference | **0,43** | **0,37** | **0,36** | **0,21** |
|  | height | 0,17 | 0,16 | 0,11 | 0,05 |
|  | Ratio WC/H | 0,32 | 0,27 | 0,29 | 0,17 |

**Figure S2**: Univariable relationship between maternal and neonatal anthropometric measures (univariable GAMs).


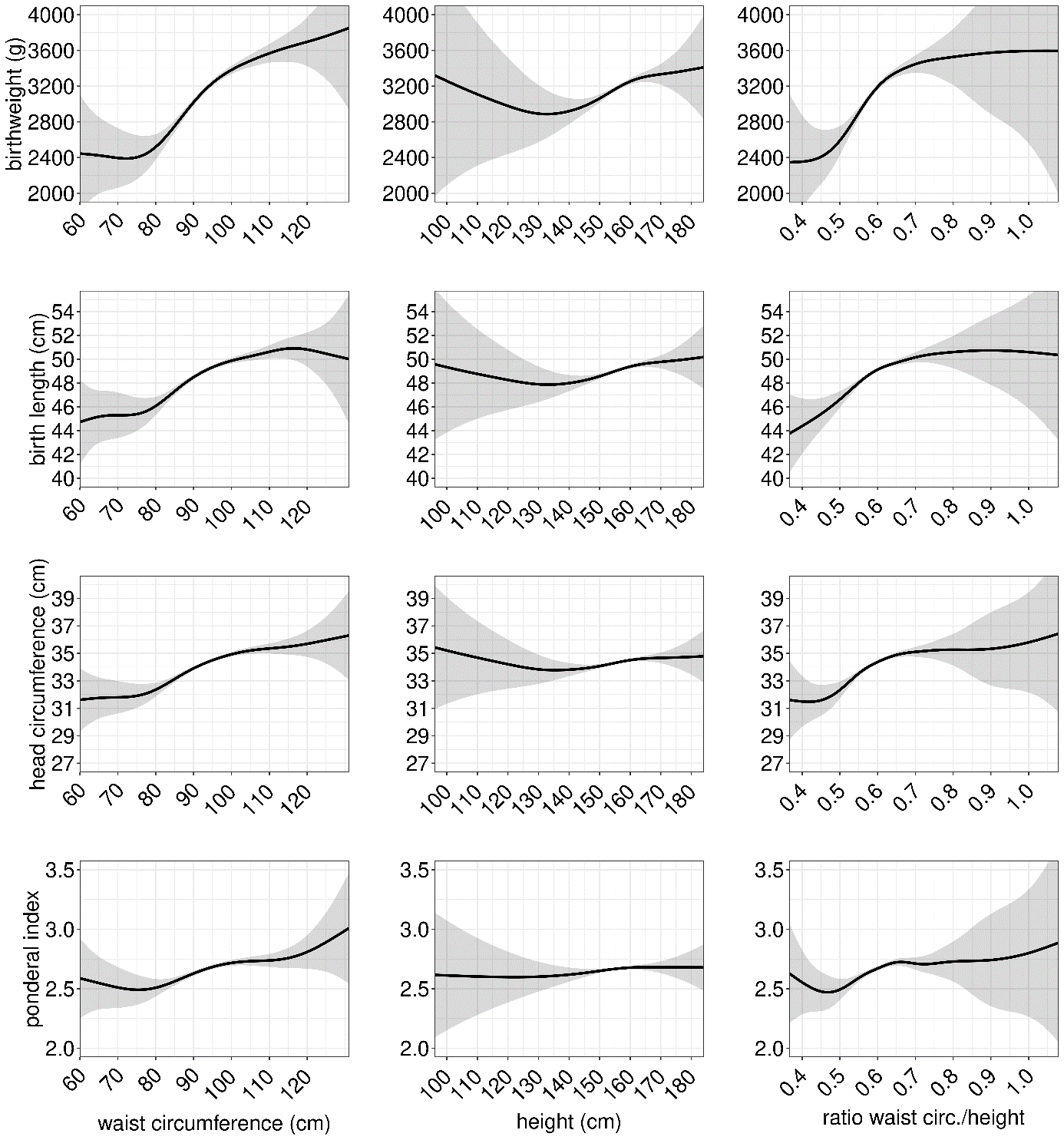


**Figure S3:** Relationship between waist circumference and maternal morphology.


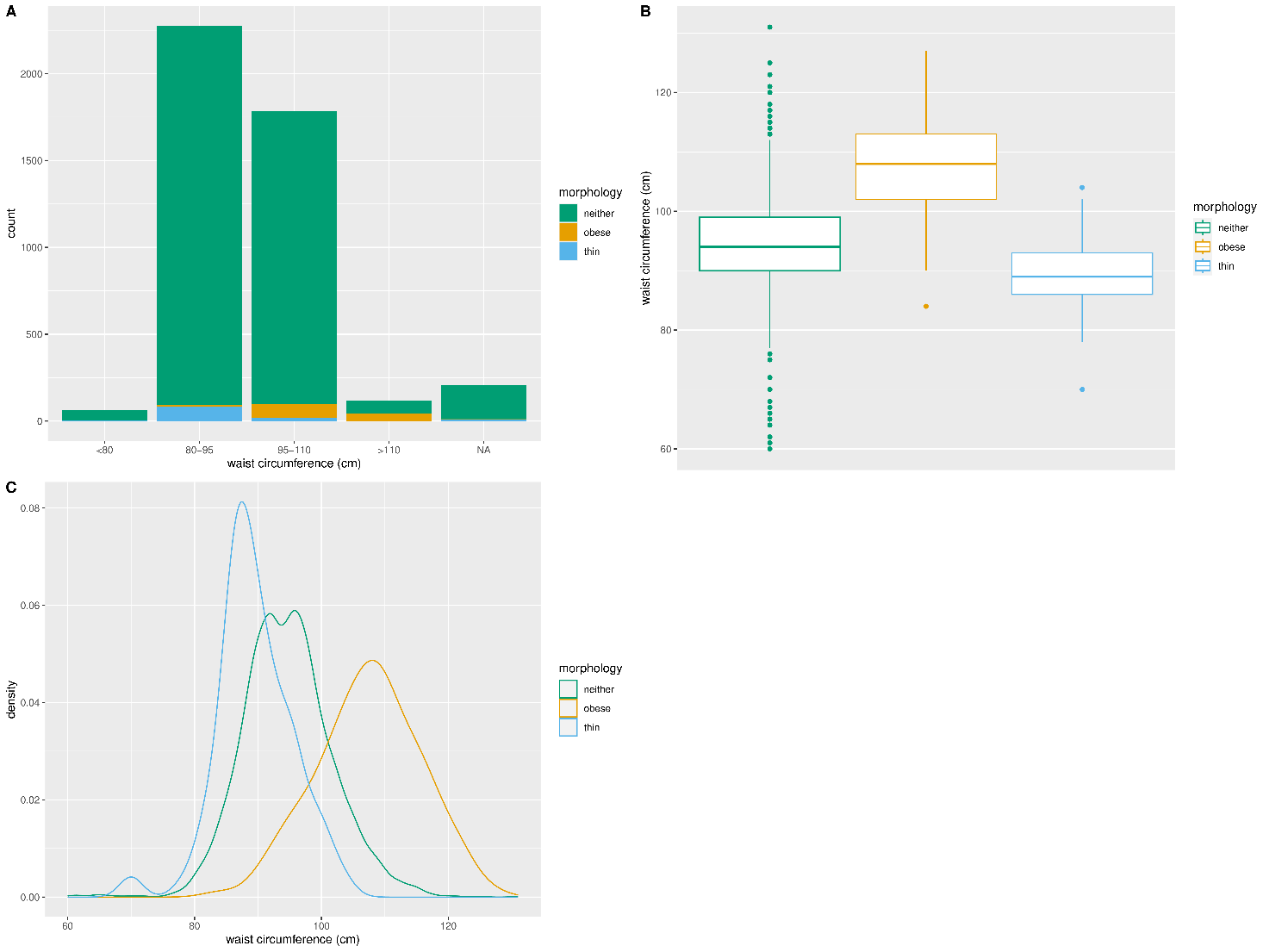


**Figure S4:** Missingness pattern. Note: HISCO class, Lausanne and civil status have their own “missing” categories.


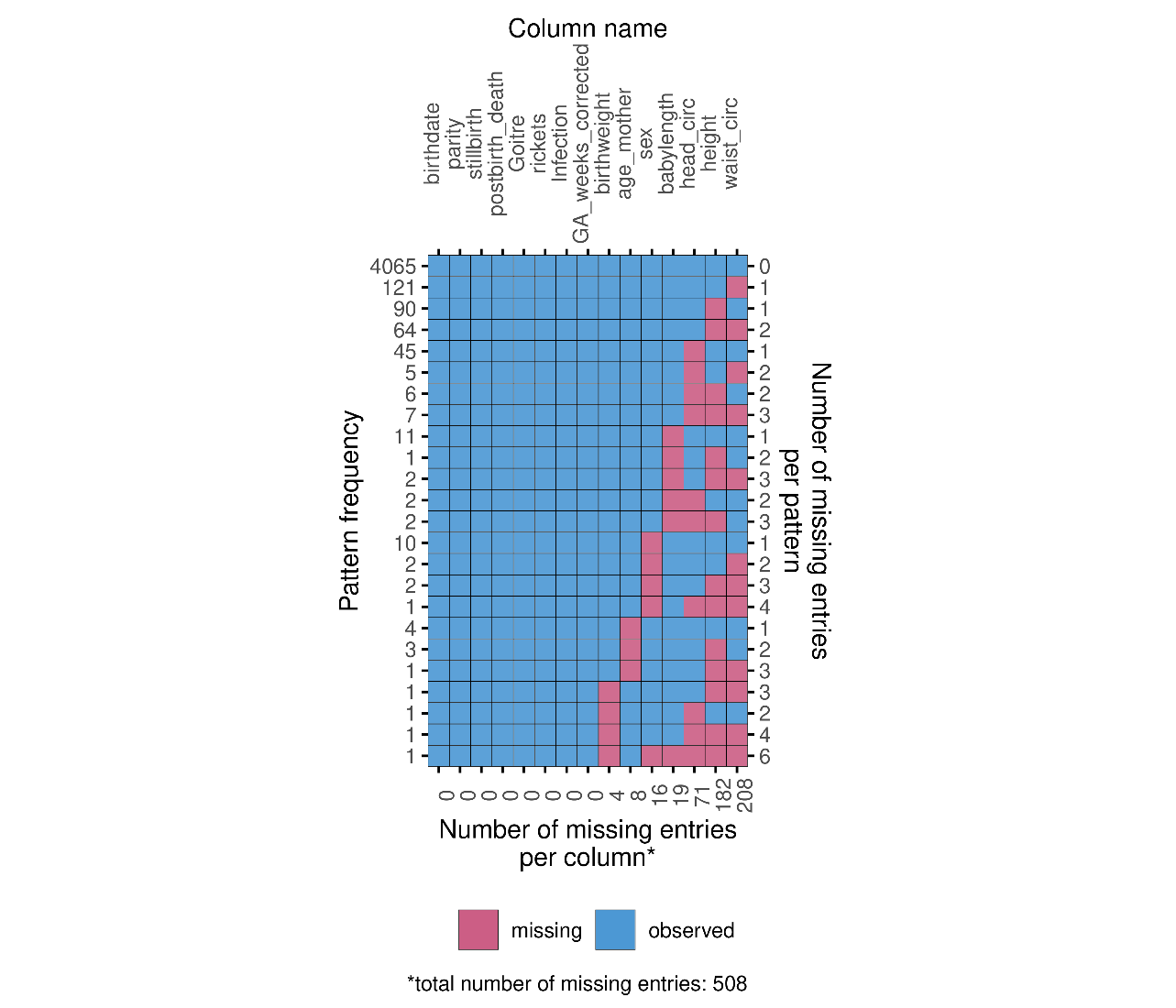


**Table S2**: Neonatal outcomes depending on maternal waist circumference, before MICE. *^1^*Mean (SD); n (%). *^2^*Wilcoxon rank sum test; Pearson's Chi-squared test; Fisher's exact test (comparing each waist circumference category with the category 80-95cm).

| **Variable** | **Waist circumference (cm)** | | | | | | |
| --- | --- | --- | --- | --- | --- | --- | --- |
|  | **<80**, N = 62 | | **80-95**, N = 2,276 *(reference)* | **95-110**, N = 1,785 | | **>110**, N = 117 | |
|  | value*^1^* | p-value*^2^* | value*^1^* | value*^1^* | p-value*^2^* | value*^1^* | p-value*^2^* |
| birth weight (g) | 2,470.5 (702.5) | <0.001 | 3,039.1 (514.8) | 3,379.3 (486.7) | <0.001 | 3,671.5 (516.8) | <0.001 |
| missing | 0 |  | 0 | 0 |  | 1 |  |
| head circumference (cm) | 32.1 (3.1) | <0.001 | 34.0 (1.7) | 35.0 (1.6) | <0.001 | 35.7 (1.4) | <0.001 |
| missing | 2 |  | 23 | 26 |  | 5 |  |
| birth length (cm) | 45.9 (4.1) | <0.001 | 48.5 (2.6) | 49.9 (2.1) | <0.001 | 51.0 (2.0) | <0.001 |
| missing | 1 |  | 7 | 7 |  | 1 |  |
| sex |  | 0.7 |  |  | <0.001 |  | 0.052 |
| male | 32 (52%) |  | 1,110 (49%) | 984 (55%) |  | 68 (58%) |  |
| female | 30 (48%) |  | 1,160 (51%) | 797 (45%) |  | 49 (42%) |  |
| missing | 0 |  | 6 | 4 |  | 0 |  |
| gestational age (weeks) |  | <0.001 |  |  | <0.001 |  | <0.001 |
| <33 | 9 (15%) |  | 61 (3%) | 21 (1%) |  | 0 (0%) |  |
| 33-37 | 19 (31%) |  | 201 (9%) | 53 (3%) |  | 0 (0%) |  |
| 37-41 | 33 (53%) |  | 1,946 (86%) | 1,560 (87%) |  | 94 (80%) |  |
| >41 | 1 (2%) |  | 68 (3%) | 151 (8%) |  | 23 (20%) |  |
| stillbirth | 5 (8%) | 0.038 | 66 (3%) | 57 (3%) | 0.6 | 7 (6%) | 0.087 |
| neonatal death d1-5 | 4 (6%) | 0.059 | 52 (2%) | 31 (2%) | 0.2 | 3 (3%) | 0.7 |
| missing | 0 |  | 3 | 1 |  | 0 |  |
| preterm birth (<37 weeks) | 28 (45%) | <0.001 | 262 (12%) | 74 (4%) | <0.001 | 0 (0%) | <0.001 |
| low birth weight (<2,500g) | 27 (44%) | <0.001 | 255 (11%) | 60 (3%) | <0.001 | 3 (3%) | 0.004 |
| missing | 0 |  | 0 | 0 |  | 1 |  |
| macrosomia (>95th percentile) | 1 (2%) | 0.6 | 31 (1%) | 142 (8%) | <0.001 | 27 (23%) | <0.001 |
| missing | 0 |  | 0 | 0 |  | 1 |  |
| ponderal index | 2.49 (0.37) | <0.001 | 2.64 (0.25) | 2.71 (0.26) |  | 2.75 (0.28) | <0.001 |
| missing | 1 |  | 7 | 7 | <0.001 | 2 |  |
| microcephaly (Z-score <-2 sd) | 14 (23%) | <0.001 | 71 (3%) | 16 (1%) | <0.001 | 0 (0%) | 0.048 |
| missing | 2 |  | 23 | 26 |  | 5 |  |

**Figure S5:** Simple directed acyclic graphs displaying the relationship between influenza and neonatal size (for instance, birth weight). Gestational age is not adjusted for and a confounding path is open (A); gestational age is adjusted for: the biasing path between exposure and outcome mediated by gestational age is closed (B); gestational age and maternal weight are adjusted for and no biasing path is open (C); gestational age is adjusted for but maternal weight is not: a colliding path is open (D); neither gestational age nor maternal weight are adjusted for: only the confounding path is open (E). Made with DAGitty v3.1 (Textor et al., 2016).

**
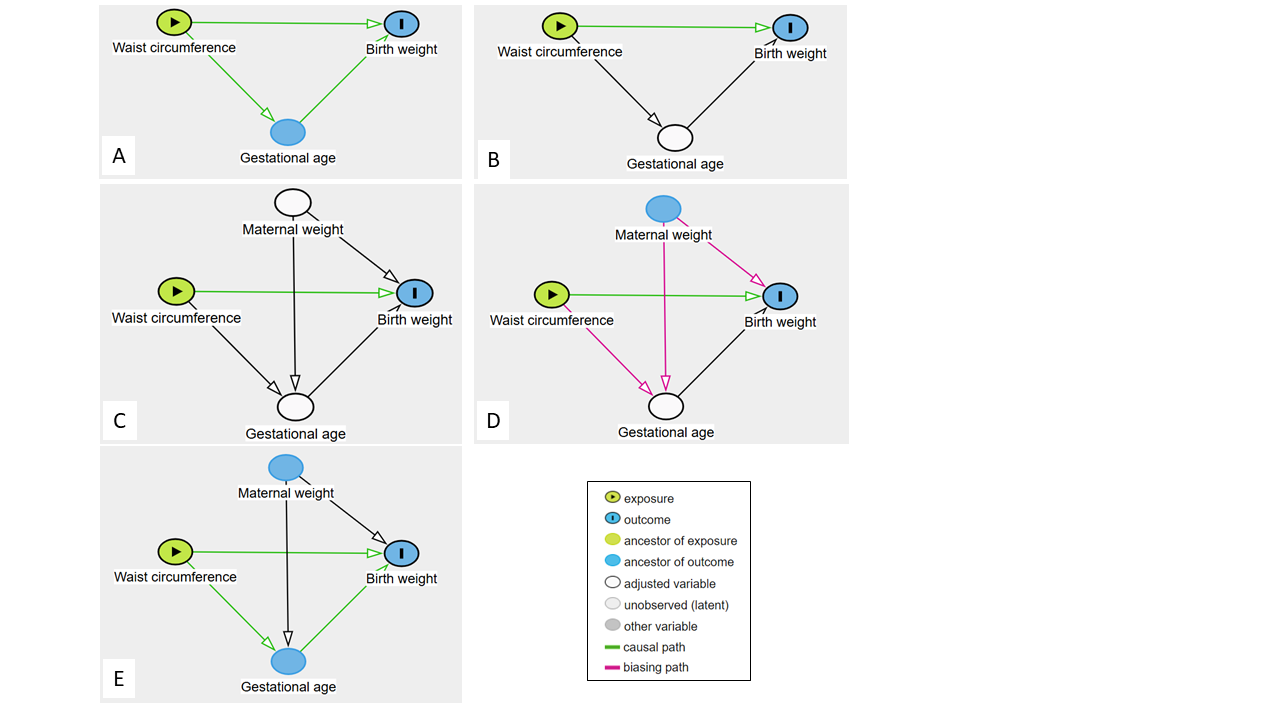
**

**Table S3**: **Relationship between waist circumference and infant size.** These are results from univariable GLMs ran on complete case and imputed datasets.

|  |  |  | Complete case (initial dataset) | | | | Imputed datasets, pooled results | | | |
| --- | --- | --- | --- | --- | --- | --- | --- | --- | --- | --- |
|  |  |  |  | 95% CI | |  |  | 95% CI | |  |
|  | Variable | Category | beta | lci | uci | p_value | beta | lci | uci | p_value |
| Birth weight (g) | (Intercept) |  | 3039.14 | 3018.34 | 3059.95 | <0.0001 | 3020.35 | 2999.09 | 3041.61 | <0.0001 |
|  | waist circumference (ref: 80-95cm) | <80cm | -568.66 | -696.44 | -440.88 | <0.0001 | -590.85 | -723.08 | -458.62 | <0.0001 |
|  |  | 95-110cm | 340.17 | 308.79 | 371.56 | <0.0001 | 354.19 | 321.58 | 386.79 | <0.0001 |
|  |  | >110cm | 632.32 | 537.83 | 726.81 | <0.0001 | 649.83 | 552.49 | 747.17 | <0.0001 |
| Head circumference (cm) | (Intercept) |  | 34.00 | 33.93 | 34.07 | <0.0001 | 33.90 | 33.83 | 33.97 | <0.0001 |
|  | waist circumference  (ref: 80-95cm) | <80cm | -1.92 | -2.34 | -1.49 | <0.0001 | -1.95 | -2.41 | -1.49 | <0.0001 |
|  |  | 95-110cm | 0.96 | 0.86 | 1.07 | <0.0001 | 1.00 | 0.89 | 1.11 | <0.0001 |
|  |  | >110cm | 1.72 | 1.40 | 2.03 | <0.0001 | 1.71 | 1.38 | 2.05 | <0.0001 |
|  | (Intercept) |  | 48.54 | 48.44 | 48.64 | <0.0001 | 48.46 | 48.35 | 48.56 | <0.0001 |
| Birth length (cm) | waist circumference  (ref: 80-95cm) | <80cm | -2.61 | -3.23 | -1.99 | <0.0001 | -2.86 | -3.53 | -2.19 | <0.0001 |
|  |  | 95-110cm | 1.33 | 1.18 | 1.48 | <0.0001 | 1.39 | 1.23 | 1.55 | <0.0001 |
|  |  | >110cm | 2.44 | 1.99 | 2.89 | <0.0001 | 2.52 | 2.06 | 2.99 | <0.0001 |
|  | (Intercept) |  | 2.64 | 2.62 | 2.65 | <0.0001 | 2.63 | 2.62 | 2.64 | <0.0001 |
| Ponderal index | waist circumference  (ref: 80-95cm) | <80cm | -0.14 | -0.21 | -0.08 | <0.001 | -0.15 | -0.21 | -0.08 | <0.001 |
|  |  | 95-110cm | 0.08 | 0.06 | 0.10 | <0.0001 | 0.08 | 0.07 | 0.10 | <0.0001 |
|  |  | >110cm | 0.12 | 0.07 | 0.17 | <0.0001 | 0.13 | 0.08 | 0.18 | <0.0001 |

**Table S4**: **Relationship between waist circumference and birth weight.** These are results from multivariable GLMs ran on complete case and imputed datasets.

|  |  | Complete case (initial dataset) | | | | Imputed datasets, pooled results | | | |
| --- | --- | --- | --- | --- | --- | --- | --- | --- | --- |
|  |  |  | 95% CI | |  |  | 95% CI | |  |
| Variable | Category | beta (g) | lci (g) | uci (g) | p_value | beta (g) | lci (g) | uci (g) | p_value |
| (Intercept) |  | 3246.06 | 3151.87 | 3340.25 | <0.0001 | 3248.68 | 3153.66 | 3343.70 | <0.0001 |
| waist circumference (ref: 80-95cm) | <80cm | -561.21 | -687.02 | -435.40 | <0.0001 | -575.17 | -706.18 | -444.17 | <0.0001 |
|  | 95-110cm | 322.56 | 290.67 | 354.45 | <0.0001 | 337.85 | 304.66 | 371.04 | <0.0001 |
|  | >110cm | 600.65 | 505.71 | 695.58 | <0.0001 | 623.42 | 526.14 | 720.70 | <0.0001 |
| maternal age (year) |  | -7.96 | -10.92 | -4.99 | <0.0001 | -8.29 | -11.27 | -5.31 | <0.0001 |
| civil status (ref: married) | single or missing | -53.63 | -101.84 | -5.43 | 0.03 | -69.77 | -118.28 | -21.26 | <0.01 |
| Lausanne (ref: no) | unsure | -1.65 | -128.66 | 125.37 | 0.98 | -17.68 | -145.83 | 110.46 | 0.79 |
|  | yes | -65.13 | -96.77 | -33.49 | <0.001 | -67.28 | -99.04 | -35.52 | <0.001 |
| year of birth (ref: 1917) | 1918 | -5.87 | -58.37 | 46.64 | 0.83 | -18.63 | -71.46 | 34.21 | 0.49 |
|  | 1919 | 17.40 | -35.23 | 70.04 | 0.52 | 1.74 | -51.03 | 54.52 | 0.95 |
|  | 1920 | 9.58 | -39.23 | 58.39 | 0.70 | 9.38 | -39.71 | 58.46 | 0.71 |
|  | 1921 | 26.64 | -21.07 | 74.34 | 0.27 | 26.32 | -21.66 | 74.29 | 0.28 |
| season (ref: spring) | summer | 23.09 | -19.00 | 65.18 | 0.28 | 10.73 | -31.61 | 53.08 | 0.62 |
|  | autumn | 62.80 | 20.19 | 105.42 | <0.01 | 64.77 | 21.81 | 107.72 | <0.01 |
|  | winter | 25.67 | -17.18 | 68.52 | 0.24 | 21.99 | -21.21 | 65.19 | 0.32 |
| parity (ref: 1) | 2 | 101.83 | 60.26 | 143.40 | <0.0001 | 98.56 | 56.83 | 140.29 | <0.0001 |
|  | >2 | 144.42 | 103.36 | 185.48 | <0.0001 | 137.42 | 96.13 | 178.70 | <0.0001 |
| sex (ref: male) | female | -102.73 | -132.91 | -72.55 | <0.0001 | -96.27 | -127.00 | -65.55 | <0.0001 |
| Infection (ref: no) | yes | -83.20 | -135.21 | -31.19 | <0.01 | -95.90 | -148.39 | -43.41 | <0.001 |

**Table S5**: **Relationship between waist circumference and head circumference.** These are results from multivariable GLMs ran on complete case and imputed datasets.

|  |  | Complete case (initial dataset) | | | | Imputed datasets, pooled results | | | |
| --- | --- | --- | --- | --- | --- | --- | --- | --- | --- |
|  |  |  | 95% CI | |  |  | 95% CI | |  |
| Variable | Category | beta (cm) | lci (cm) | uci (cm) | p_value | beta (cm) | lci (cm) | uci (cm) | p_value |
| (Intercept) |  | 34.74 | 34.43 | 35.05 | <0.0001 | 34.85 | 34.52 | 35.17 | <0.0001 |
| waist circumference (ref: 80-95cm) | <80cm | -1.93 | -2.35 | -1.52 | <0.0001 | -1.93 | -2.38 | -1.47 | <0.0001 |
|  | 95-110cm | 0.94 | 0.84 | 1.04 | <0.0001 | 0.99 | 0.88 | 1.10 | <0.0001 |
|  | >110cm | 1.74 | 1.42 | 2.05 | <0.0001 | 1.76 | 1.42 | 2.09 | <0.0001 |
| maternal age (year) |  | -0.01 | -0.02 | 0.00 | <0.01 | -0.02 | -0.03 | -0.01 | <0.001 |
| civil status (ref: married) | single or missing | -0.23 | -0.39 | -0.07 | <0.01 | -0.34 | -0.51 | -0.17 | <0.001 |
| Lausanne (ref: no) | unsure | -0.12 | -0.53 | 0.29 | 0.56 | -0.14 | -0.58 | 0.29 | 0.52 |
|  | yes | -0.30 | -0.40 | -0.19 | <0.0001 | -0.34 | -0.45 | -0.23 | <0.0001 |
| year of birth (ref: 1917) | 1918 | 0.17 | -0.01 | 0.34 | 0.06 | 0.16 | -0.02 | 0.34 | 0.09 |
|  | 1919 | 0.06 | -0.12 | 0.23 | 0.52 | 0.04 | -0.14 | 0.22 | 0.65 |
|  | 1920 | -0.14 | -0.30 | 0.02 | 0.08 | -0.13 | -0.30 | 0.04 | 0.13 |
|  | 1921 | 0.06 | -0.10 | 0.21 | 0.48 | 0.06 | -0.10 | 0.23 | 0.47 |
| season (ref: spring) | summer | 0.02 | -0.12 | 0.16 | 0.75 | -0.05 | -0.19 | 0.10 | 0.54 |
|  | autumn | 0.09 | -0.05 | 0.23 | 0.19 | 0.11 | -0.04 | 0.26 | 0.15 |
|  | winter | 0.21 | 0.07 | 0.35 | <0.01 | 0.15 | 0.00 | 0.30 | 0.05 |
| parity (ref: 1) | 2 | 0.05 | -0.09 | 0.19 | 0.46 | 0.05 | -0.09 | 0.19 | 0.49 |
|  | >2 | -0.07 | -0.20 | 0.07 | 0.32 | -0.10 | -0.24 | 0.04 | 0.17 |
| sex (ref: male) | female | -0.56 | -0.66 | -0.46 | <0.0001 | -0.56 | -0.67 | -0.46 | <0.0001 |
| Infection (ref: no) | yes | -0.36 | -0.53 | -0.19 | <0.001 | -0.40 | -0.58 | -0.22 | <0.001 |

**Table S6**: **Relationship between waist circumference and birth length.** These are results from multivariable GLMs ran on complete case and imputed datasets.

|  |  | Complete case (initial dataset) | | | | Imputed datasets, pooled results | | | |
| --- | --- | --- | --- | --- | --- | --- | --- | --- | --- |
|  |  |  | 95% CI | |  |  | 95% CI | |  |
| Variable | Category | beta (cm) | lci (cm) | uci (cm) | p_value | beta (cm) | lci (cm) | uci (cm) | p_value |
| (Intercept) |  | 49.31 | 48.86 | 49.76 | <0.0001 | 49.36 | 48.90 | 49.82 | <0.0001 |
| waist circumference (ref: 80-95cm) | <80cm | -2.58 | -3.19 | -1.98 | <0.0001 | -2.81 | -3.47 | -2.15 | <0.0001 |
|  | 95-110cm | 1.25 | 1.09 | 1.40 | <0.0001 | 1.32 | 1.16 | 1.48 | <0.0001 |
|  | >110cm | 2.30 | 1.85 | 2.76 | <0.0001 | 2.41 | 1.94 | 2.88 | <0.0001 |
| maternal age (year) |  | -0.01 | -0.03 | 0.00 | 0.08 | -0.02 | -0.03 | 0.00 | 0.04 |
| civil status (ref: married) | single or missing | -0.33 | -0.56 | -0.10 | <0.01 | -0.37 | -0.61 | -0.14 | <0.01 |
| Lausanne (ref: no) | unsure | -0.13 | -0.74 | 0.48 | 0.67 | -0.16 | -0.78 | 0.47 | 0.63 |
|  | yes | -0.43 | -0.58 | -0.27 | <0.0001 | -0.43 | -0.59 | -0.28 | <0.0001 |
| year of birth (ref: 1917) | 1918 | 0.01 | -0.24 | 0.26 | 0.93 | -0.08 | -0.34 | 0.18 | 0.55 |
|  | 1919 | 0.05 | -0.20 | 0.30 | 0.70 | -0.06 | -0.32 | 0.20 | 0.64 |
|  | 1920 | -0.15 | -0.39 | 0.08 | 0.20 | -0.17 | -0.41 | 0.07 | 0.15 |
|  | 1921 | 0.02 | -0.21 | 0.25 | 0.89 | 0.02 | -0.22 | 0.25 | 0.88 |
| season (ref: spring) | summer | 0.07 | -0.13 | 0.27 | 0.49 | 0.01 | -0.20 | 0.21 | 0.95 |
|  | autumn | 0.23 | 0.02 | 0.43 | 0.03 | 0.24 | 0.03 | 0.45 | 0.03 |
|  | winter | 0.06 | -0.14 | 0.27 | 0.54 | 0.03 | -0.18 | 0.24 | 0.78 |
| parity (ref: 1) | 2 | 0.05 | -0.15 | 0.25 | 0.64 | 0.02 | -0.19 | 0.22 | 0.86 |
|  | >2 | 0.20 | 0.00 | 0.40 | 0.05 | 0.18 | -0.02 | 0.38 | 0.08 |
| sex (ref: male) | female | -0.58 | -0.72 | -0.43 | <0.0001 | -0.56 | -0.71 | -0.41 | <0.0001 |
| Infection (ref: no) | yes | -0.42 | -0.67 | -0.17 | <0.001 | -0.44 | -0.70 | -0.19 | <0.001 |

**Table S7**: **Relationship between waist circumference and ponderal index.** These are results from multivariable GLMs ran on complete case and imputed datasets.

|  |  | Complete case (initial dataset) | | | | Imputed datasets, pooled results | | | |
| --- | --- | --- | --- | --- | --- | --- | --- | --- | --- |
|  |  |  | 95% CI | |  |  | 95% CI | |  |
| Variable | Category | beta | lci | uci | p_value | beta | lci | uci | p_value |
| (Intercept) |  | 2.72 | 2.67 | 2.77 | <0.0001 | 2.71 | 2.66 | 2.76 | <0.0001 |
| waist circumference (ref: 80-95cm) | <80cm | -0.14 | -0.21 | -0.08 | <0.001 | -0.14 | -0.21 | -0.07 | <0.001 |
|  | 95-110cm | 0.08 | 0.06 | 0.10 | <0.0001 | 0.08 | 0.07 | 0.10 | <0.0001 |
|  | >110cm | 0.12 | 0.07 | 0.17 | <0.0001 | 0.14 | 0.08 | 0.19 | <0.0001 |
| maternal age (year) |  | -0.01 | -0.01 | 0.00 | <0.0001 | -0.01 | -0.01 | 0.00 | <0.0001 |
| civil status (ref: married) | single or missing | 0.00 | -0.03 | 0.02 | 0.78 | -0.01 | -0.04 | 0.01 | 0.33 |
| Lausanne (ref: no) | unsure | 0.03 | -0.04 | 0.09 | 0.43 | 0.02 | -0.05 | 0.08 | 0.60 |
|  | yes | 0.01 | -0.01 | 0.02 | 0.31 | 0.01 | -0.01 | 0.02 | 0.32 |
| year of birth (ref: 1917) | 1918 | -0.01 | -0.04 | 0.02 | 0.51 | -0.01 | -0.03 | 0.02 | 0.58 |
|  | 1919 | 0.00 | -0.03 | 0.03 | 0.96 | 0.00 | -0.03 | 0.03 | 0.97 |
|  | 1920 | 0.03 | 0.00 | 0.05 | 0.05 | 0.03 | 0.00 | 0.05 | 0.02 |
|  | 1921 | 0.01 | -0.01 | 0.04 | 0.25 | 0.02 | -0.01 | 0.04 | 0.17 |
| season (ref: spring) | summer | 0.00 | -0.03 | 0.02 | 0.70 | -0.01 | -0.03 | 0.02 | 0.56 |
|  | autumn | 0.01 | -0.01 | 0.04 | 0.22 | 0.02 | -0.01 | 0.04 | 0.17 |
|  | winter | 0.01 | -0.02 | 0.03 | 0.57 | 0.01 | -0.02 | 0.03 | 0.63 |
| parity (ref: 1) | 2 | 0.07 | 0.05 | 0.10 | <0.0001 | 0.08 | 0.06 | 0.10 | <0.0001 |
|  | >2 | 0.09 | 0.06 | 0.11 | <0.0001 | 0.08 | 0.06 | 0.10 | <0.0001 |
| sex (ref: male) | female | 0.01 | 0.00 | 0.03 | 0.08 | 0.01 | 0.00 | 0.03 | 0.07 |
| Infection (ref: no) | yes | -0.01 | -0.04 | 0.02 | 0.51 | -0.01 | -0.04 | 0.01 | 0.32 |

**Table S8**: **Relationship between waist circumference and binary pregnancy outcomes.** These are results from univariable logistic GLMs ran on complete case and imputed datasets.

|  |  |  | Complete case (initial dataset) | | | | Imputed datasets, pooled results | | | |
| --- | --- | --- | --- | --- | --- | --- | --- | --- | --- | --- |
|  |  |  |  | 95% CI | |  |  | 95% CI | |  |
| Outcome variable | Variable | Category | OR | lci | uci | p_value | OR | lci | uci | p_value |
| Low birth weight (<2,500g) | (Intercept) |  | 0.13 | 0.11 | 0.14 | <0.0001 | 0.14 | 0.12 | 0.16 | <0.0001 |
|  | waist circumference  (ref: 80-95cm) | <80cm | 6.11 | 3.64 | 10.27 | <0.0001 | 5.93 | 3.59 | 9.79 | <0.0001 |
|  |  | 95-110cm | 0.28 | 0.21 | 0.37 | <0.0001 | 0.26 | 0.19 | 0.34 | <0.0001 |
|  |  | >110cm | 0.21 | 0.07 | 0.67 | <0.01 | 0.19 | 0.06 | 0.59 | <0.01 |
|  | (Intercept) |  | 0.13 | 0.11 | 0.15 | <0.0001 | 0.14 | 0.13 | 0.16 | <0.0001 |
| Preterm birth (<37 weeks) | waist circumference  (ref: 80-95cm) | <80cm | 6.33 | 3.78 | 10.61 | <0.0001 | 5.96 | 3.57 | 9.95 | <0.0001 |
|  |  | >95cm | 0.31 | 0.24 | 0.41 | <0.0001 | 0.30 | 0.23 | 0.39 | <0.0001 |
| Macrosomia (>95^th^ percentile) | (Intercept) |  | 0.01 | 0.01 | 0.02 | <0.0001 | 0.01 | 0.01 | 0.02 | <0.0001 |
|  | waist circumference  (ref: 80-95cm) | <80cm | 1.19 | 0.16 | 8.84 | 0.87 | 1.10 | 0.15 | 8.15 | 0.93 |
|  |  | 95-110cm | 6.26 | 4.22 | 9.28 | <0.0001 | 6.24 | 4.22 | 9.24 | <0.0001 |
|  |  | >110cm | 21.97 | 12.58 | 38.38 | <0.0001 | 22.48 | 12.92 | 39.13 | <0.0001 |
| Microcephaly (Z-score < 2sd) | (Intercept) |  | 0.03 | 0.03 | 0.04 | <0.0001 | 0.04 | 0.04 | 0.05 | <0.0001 |
|  | waist circumference  (ref: 80-95cm) | <80cm | 9.35 | 4.92 | 17.80 | <0.0001 | 7.49 | 4.08 | 13.75 | <0.0001 |
|  |  | >95cm | 0.27 | 0.15 | 0.46 | <0.0001 | 0.30 | 0.19 | 0.49 | <0.0001 |
| Stillbirth | (Intercept) |  | 0.03 | 0.02 | 0.04 | <0.0001 | 0.04 | 0.03 | 0.04 | <0.0001 |
|  | waist circumference  (ref: 80-95cm) | <80cm | 2.94 | 1.14 | 7.57 | 0.03 | 2.73 | 1.09 | 6.87 | 0.03 |
|  |  | 95-110cm | 1.10 | 0.77 | 1.58 | 0.59 | 1.05 | 0.74 | 1.50 | 0.79 |
|  |  | >110cm | 2.13 | 0.96 | 4.75 | 0.06 | 1.91 | 0.87 | 4.18 | 0.11 |
| Neonatal mortality day1-5 | (Intercept) |  | 0.02 | 0.02 | 0.03 | <0.0001 | 0.02 | 0.02 | 0.03 | <0.0001 |
|  | waist circumference  (ref: 80-95cm) | <80cm | 2.95 | 1.03 | 8.42 | 0.04 | 2.96 | 1.04 | 8.42 | 0.04 |
|  |  | 95-110cm | 0.76 | 0.48 | 1.18 | 0.22 | 0.69 | 0.44 | 1.07 | 0.10 |
|  |  | >110cm | 1.12 | 0.35 | 3.65 | 0.85 | 1.01 | 0.31 | 3.29 | 0.98 |

**Table S9**: **Relationship between waist circumference and low birth weight (birth weight <2,500g).** These are results from multivariable logistic GLMs ran on complete case and imputed datasets.

|  |  | Complete case (initial dataset) | | | | Imputed datasets, pooled results | | | |
| --- | --- | --- | --- | --- | --- | --- | --- | --- | --- |
|  |  |  | 95% CI | |  |  | 95% CI | |  |
| Variable | Category | beta | lci | uci | p_value | beta | lci | uci | p_value |
| (Intercept) |  | 0.02 | 0.01 | 0.05 | <0.0001 | 0.03 | 0.02 | 0.06 | <0.0001 |
| waist circumference (ref: 80-95cm) | <80cm | 6.02 | 3.52 | 10.29 | <0.0001 | 5.76 | 3.42 | 9.71 | <0.0001 |
|  | 95-110cm | 0.27 | 0.20 | 0.37 | <0.0001 | 0.25 | 0.19 | 0.34 | <0.0001 |
|  | >110cm | 0.19 | 0.06 | 0.60 | <0.01 | 0.17 | 0.05 | 0.54 | <0.01 |
| maternal age (year) |  | 1.04 | 1.02 | 1.07 | <0.001 | 1.04 | 1.02 | 1.06 | <0.001 |
| civil status (ref: married) | single or missing | 1.75 | 1.28 | 2.39 | <0.001 | 1.84 | 1.38 | 2.46 | <0.001 |
| Lausanne (ref: no) | unsure | 0.78 | 0.23 | 2.63 | 0.69 | 0.70 | 0.21 | 2.35 | 0.57 |
|  | yes | 1.43 | 1.13 | 1.80 | <0.01 | 1.41 | 1.13 | 1.75 | <0.01 |
| year of birth (ref: 1917) | 1918 | 1.14 | 0.76 | 1.71 | 0.53 | 1.18 | 0.81 | 1.71 | 0.39 |
|  | 1919 | 1.25 | 0.84 | 1.87 | 0.28 | 1.26 | 0.87 | 1.83 | 0.23 |
|  | 1920 | 1.22 | 0.83 | 1.80 | 0.30 | 1.18 | 0.83 | 1.70 | 0.36 |
|  | 1921 | 1.23 | 0.85 | 1.79 | 0.28 | 1.12 | 0.79 | 1.60 | 0.52 |
| season (ref: spring) | summer | 1.30 | 0.95 | 1.78 | 0.10 | 1.30 | 0.97 | 1.75 | 0.08 |
|  | autumn | 1.04 | 0.74 | 1.45 | 0.83 | 0.97 | 0.71 | 1.34 | 0.85 |
|  | winter | 1.15 | 0.83 | 1.60 | 0.40 | 1.12 | 0.82 | 1.52 | 0.47 |
| parity (ref: 1) | 2 | 0.79 | 0.57 | 1.09 | 0.15 | 0.78 | 0.58 | 1.06 | 0.12 |
|  | >2 | 0.84 | 0.61 | 1.14 | 0.26 | 0.90 | 0.67 | 1.20 | 0.47 |
| sex (ref: male) | female | 1.04 | 0.82 | 1.30 | 0.77 | 1.04 | 0.84 | 1.29 | 0.72 |
| Infection (ref: no) | yes | 1.85 | 1.33 | 2.57 | <0.001 | 1.87 | 1.37 | 2.55 | <0.001 |

**Table S10**: **Relationship between waist circumference and preterm birth (gestational age <37 weeks).** These are results from multivariable logistic GLMs ran on complete case and imputed datasets. Note: as there were no cases of preterm birth among mothers with a waist circumference >110cm, categories 95-110cm and >110cm were pooled together.

|  |  | Complete case (initial dataset) | | | | Imputed datasets, pooled results | | | |
| --- | --- | --- | --- | --- | --- | --- | --- | --- | --- |
|  |  |  | 95% CI | |  |  | 95% CI | |  |
| Variable | Category | beta | lci | uci | p_value | beta | lci | uci | p_value |
| (Intercept) |  | 0.08 | 0.04 | 0.17 | <0.0001 | 0.08 | 0.04 | 0.16 | <0.0001 |
| waist circumference (ref: 80-95cm) | <80cm | 6.34 | 3.72 | 10.82 | <0.0001 | 5.86 | 3.46 | 9.93 | <0.0001 |
|  | >95cm | 0.32 | 0.24 | 0.42 | <0.0001 | 0.30 | 0.23 | 0.40 | <0.0001 |
| maternal age (year) |  | 1.02 | 1.00 | 1.04 | 0.05 | 1.03 | 1.01 | 1.05 | 0.01 |
| civil status (ref: married) | single or missing | 1.69 | 1.24 | 2.29 | <0.001 | 1.74 | 1.30 | 2.32 | <0.001 |
| Lausanne (ref: no) | unsure | 0.87 | 0.26 | 2.87 | 0.82 | 0.76 | 0.23 | 2.49 | 0.64 |
|  | yes | 1.66 | 1.32 | 2.08 | <0.001 | 1.53 | 1.23 | 1.89 | <0.001 |
| year of birth (ref: 1917) | 1918 | 0.55 | 0.38 | 0.79 | <0.01 | 0.59 | 0.42 | 0.83 | <0.01 |
|  | 1919 | 0.49 | 0.34 | 0.72 | <0.001 | 0.54 | 0.38 | 0.77 | <0.001 |
|  | 1920 | 0.63 | 0.45 | 0.88 | <0.01 | 0.65 | 0.47 | 0.90 | <0.01 |
|  | 1921 | 0.55 | 0.40 | 0.77 | <0.001 | 0.55 | 0.40 | 0.76 | <0.001 |
| season (ref: spring) | summer | 0.67 | 0.49 | 0.92 | 0.01 | 0.71 | 0.53 | 0.95 | 0.02 |
|  | autumn | 0.77 | 0.56 | 1.05 | 0.10 | 0.76 | 0.57 | 1.02 | 0.07 |
|  | winter | 0.81 | 0.59 | 1.10 | 0.17 | 0.84 | 0.63 | 1.12 | 0.25 |
| parity (ref: 1) | 2 | 0.92 | 0.67 | 1.26 | 0.61 | 0.98 | 0.73 | 1.31 | 0.89 |
|  | >2 | 1.09 | 0.81 | 1.48 | 0.56 | 1.07 | 0.80 | 1.42 | 0.67 |
| sex (ref: male) | female | 1.22 | 0.97 | 1.52 | 0.09 | 1.14 | 0.92 | 1.41 | 0.24 |
| Infection (ref: no) | yes | 1.75 | 1.25 | 2.45 | <0.01 | 1.85 | 1.35 | 2.53 | <0.001 |

**Table S11**: **Marginal probabilities of binary outcomes depending on waist circumference.** These are results from multivariable logistic GLMs ran on complete case datasets. Note: as there were no cases of preterm birth and microcephaly among mothers with a waist circumference >110cm, categories 95-110cm and >110cm were pooled together. These are the same models than presented in **Table 4**.

|  |  | Univariable - absolute risk | | | Multivariable - absolute risk | | |
| --- | --- | --- | --- | --- | --- | --- | --- |
|  |  |  | 95%CI | |  | 95%CI | |
| Outcome variable | Waist circumference category (cm) | % | lci | uci | % | lci | uci |
| Low birth weight (<2,500g) | 80-95 | 11.20 | 9.97 | 12.57 | 80-95 | 14.28 | 9.63 |
|  | <80 | 43.55 | 31.83 | 56.03 | <80 | 50.05 | 34.12 |
|  | 95-110 | 3.36 | 2.62 | 4.31 | 95-110 | 4.34 | 2.68 |
|  | >110 | 2.59 | 0.84 | 7.71 | >110 | 3.02 | 0.91 |
|  | 80-95 | 11.51 | 10.26 | 12.89 | 14.51 | 9.84 | 20.87 |
| Preterm birth (<37 weeks) | <80 | 45.16 | 33.31 | 57.59 | 51.84 | 35.81 | 67.50 |
|  | >95 | 3.89 | 3.11 | 4.86 | 5.12 | 3.23 | 8.01 |
| Macrosomia (>95^th^ percentile) | 80-95 | 1.36 | 0.96 | 1.93 |  |  |  |
|  | <80 | 1.61 | 0.23 | 10.57 |  |  |  |
|  | 95-110 | 7.96 | 6.79 | 9.30 |  |  |  |
|  | >110 | 23.28 | 16.47 | 31.82 |  |  |  |
| Microcephaly (*Z*-score < 2sd) | 80-95 | 3.15 | 2.50 | 3.96 |  |  |  |
|  | <80 | 23.33 | 14.33 | 35.63 |  |  |  |
|  | >95 | 0.86 | 0.52 | 1.39 |  |  |  |
| Stillbirth | 80-95 | 2.29 | 1.75 | 2.99 |  |  |  |
|  | <80 | 6.45 | 2.44 | 15.96 |  |  |  |
|  | 95-110 | 1.74 | 1.22 | 2.46 |  |  |  |
|  | >110 | 2.56 | 0.83 | 7.65 |  |  |  |
| Neonatal mortality day1-5 | 80-95 | 2.29 | 1.75 | 2.99 |  |  |  |
|  | <80 | 6.45 | 2.44 | 15.96 |  |  |  |
|  | 95-110 | 1.74 | 1.22 | 2.46 |  |  |  |
|  | >110 | 2.56 | 0.83 | 7.65 |  |  |  |

**Sensitivity analyses: Primiparous women**

**Table S12**: **Sensitivity analysis on primiparous women only: relationship between waist circumference and infant size.** These are pooled results from univariable and multivariable GLMs ran on imputed datasets. Multivariable GLMs are adjusted for maternal age, civil status, living in Lausanne, birth, birth season, infection and infant sex.

|  |  |  | Univariable | | | | Multivariable | | | |
| --- | --- | --- | --- | --- | --- | --- | --- | --- | --- | --- |
|  |  |  |  | 95% CI | |  |  | 95% CI | |  |
|  | Variable | Category | beta | lci | uci | p_value | beta | lci | uci | p_value |
| Birth weight (g) | (Intercept) |  | 2982.33 | 2953.76 | 3010.90 | <0.0001 | 3303.32 | 3150.43 | 3456.21 | <0.0001 |
|  | waist circumference  (ref: 80-95cm) | <80cm | -583.72 | -753.45 | -413.99 | <0.0001 | -576.81 | -745.91 | -407.71 | <0.0001 |
|  |  | 95-110cm | 307.65 | 259.05 | 356.24 | <0.0001 | 299.69 | 250.83 | 348.54 | <0.0001 |
|  |  | >110cm | 545.40 | 233.96 | 856.83 | <0.001 | 533.27 | 223.70 | 842.84 | <0.001 |
|  | (Intercept) |  | 33.92 | 33.81 | 34.03 | <0.0001 | 35.10 | 34.53 | 35.66 | <0.0001 |
| Head circumference (cm) | waist circumference  (ref: 80-95cm) | <80cm | -2.12 | -2.77 | -1.47 | <0.0001 | -2.07 | -2.71 | -1.43 | <0.0001 |
|  |  | 95-110cm | 1.04 | 0.86 | 1.22 | <0.0001 | 1.00 | 0.82 | 1.17 | <0.0001 |
|  |  | >110cm | 2.06 | 0.90 | 3.22 | <0.001 | 1.96 | 0.82 | 3.09 | <0.001 |
| Birth length (cm) | (Intercept) |  | 48.42 | 48.27 | 48.57 | <0.0001 | 49.32 | 48.52 | 50.12 | <0.0001 |
|  | waist circumference  (ref: 80-95cm) | <80cm | -2.87 | -3.78 | -1.97 | <0.0001 | -2.79 | -3.69 | -1.88 | <0.0001 |
|  |  | 95-110cm | 1.28 | 1.02 | 1.53 | <0.0001 | 1.21 | 0.95 | 1.46 | <0.0001 |
|  |  | >110cm | 2.41 | 0.73 | 4.09 | <0.01 | 2.19 | 0.53 | 3.86 | 0.01 |
| Ponderal index | (Intercept) |  | 2.61 | 2.59 | 2.62 | <0.0001 | 2.77 | 2.69 | 2.85 | <0.0001 |
|  | waist circumference  (ref: 80-95cm) | <80cm | -0.15 | -0.24 | -0.06 | <0.001 | -0.16 | -0.25 | -0.07 | <0.001 |
|  |  | 95-110cm | 0.07 | 0.04 | 0.09 | <0.0001 | 0.07 | 0.05 | 0.10 | <0.0001 |
|  |  | >110cm | 0.10 | -0.07 | 0.26 | 0.25 | 0.12 | -0.04 | 0.29 | 0.15 |

**Table S13**: **Sensitivity analysis on primiparous women only: relationship between waist circumference and binary pregnancy outcomes**. These are pooled results from univariable and multivariable logistic GLMs ran on imputed datasets. Multivariable GLMs are adjusted for maternal age, civil status, living in Lausanne, birth, birth season, infection and infant sex. Due to the low number of events, waist circumference 95-110cm and >110cm were pooled together, and the macrosomia outcome GLM was not run.

|  |  |  | Univariable | | | | Multivariable | | | |
| --- | --- | --- | --- | --- | --- | --- | --- | --- | --- | --- |
|  |  |  |  | 95% CI | |  |  | 95% CI | |  |
| Outcome variable | Variable | Category | OR | lci | uci | p_value | OR | lci | uci | p_value |
| Low birth weight (<2,500g) | (Intercept) |  | 0.15 | 0.12 | 0.18 | <0.0001 | 0.05 | 0.02 | 0.15 | <0.0001 |
|  | waist circumference  (ref: 80-95cm) | <80cm | 5.85 | 2.89 | 11.85 | <0.0001 | 5.23 | 2.52 | 10.87 | <0.001 |
|  |  | >95cm | 0.24 | 0.15 | 0.39 | <0.0001 | 0.26 | 0.16 | 0.42 | <0.0001 |
| Preterm birth (<37 weeks) | (Intercept) |  | 0.14 | 0.12 | 0.17 | <0.0001 | 0.15 | 0.05 | 0.44 | <0.001 |
|  | waist circumference  (ref: 80-95cm) | <80cm | 6.89 | 3.43 | 13.83 | <0.0001 | 7.20 | 3.44 | 15.06 | <0.0001 |
|  |  | >95cm | 0.30 | 0.19 | 0.47 | <0.0001 | 0.34 | 0.21 | 0.54 | <0.0001 |
| Microcephaly (Z-score < 2sd) | (Intercept) |  | 0.04 | 0.03 | 0.06 | <0.0001 | - | | | |
|  | waist circumference  (ref: 80-95cm) | <80cm | 9.09 | 4.02 | 20.54 | <0.0001 |  |  |  |  |
|  |  | >95cm | 0.10 | 0.03 | 0.43 | <0.01 |  |  |  |  |
| Stillbirth | (Intercept) |  | 0.04 | 0.03 | 0.05 | <0.0001 | - | | | |
|  | waist circumference  (ref: 80-95cm) | <80cm | 1.93 | 0.41 | 8.97 | 0.40 |  |  |  |  |
|  |  | >95cm | 0.88 | 0.49 | 1.60 | 0.68 |  |  |  |  |
| Neonatal mortality day1-5 | (Intercept) |  | 0.02 | 0.01 | 0.03 | <0.0001 | - | | | |
|  | waist circumference  (ref: 80-95cm) | <80cm | 3.69 | 0.84 | 16.30 | 0.09 |  |  |  |  |
|  |  | >95cm | 0.89 | 0.43 | 1.85 | 0.76 |  |  |  |  |

**Figure S6**: effect of waist circumference (modelled using a spline) on neonatal anthropometrics, using the imputed datasets. The reference waist circumference is 95cm (median of the sample). Shaded band = 95% CI.


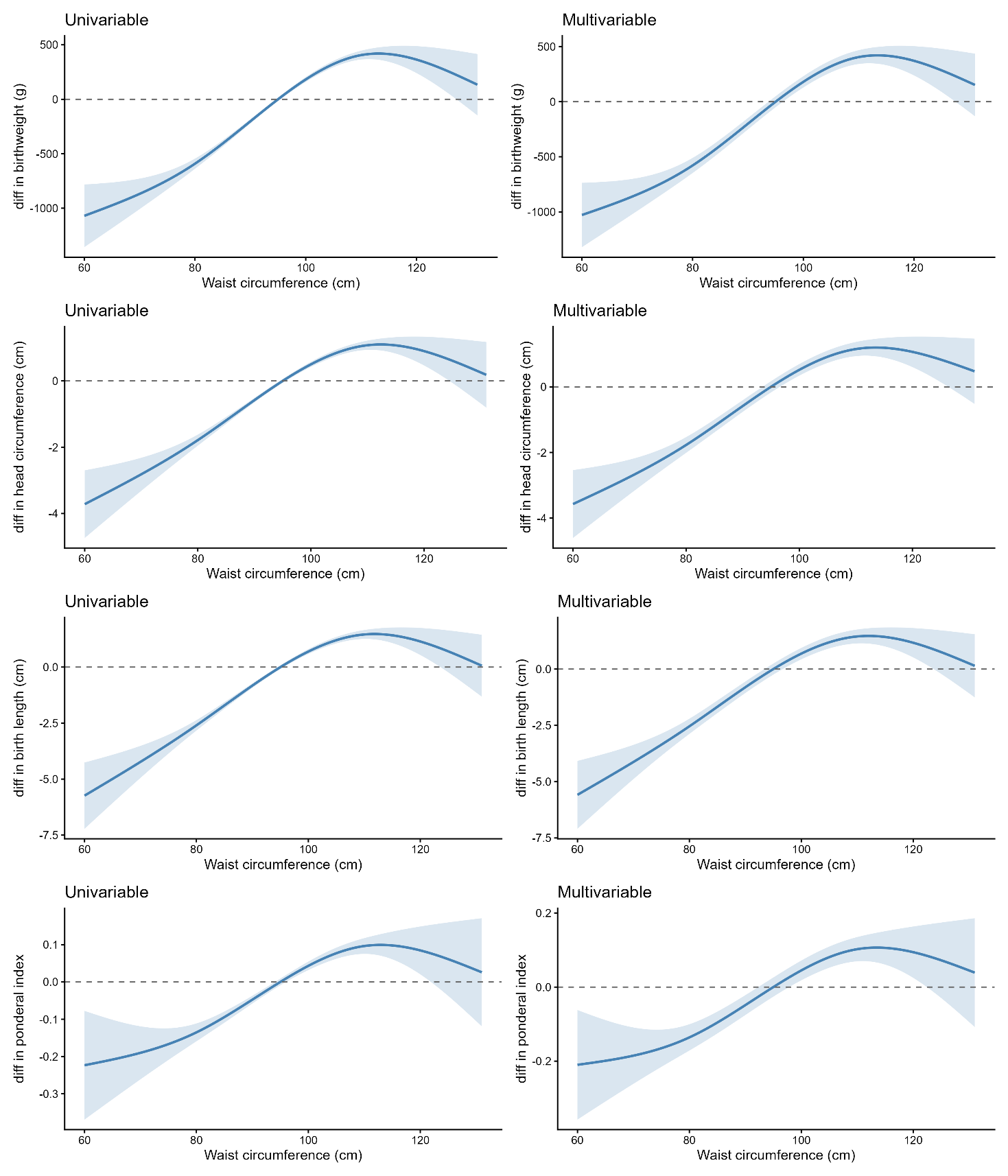


**Table S14**: effect of waist circumference (modelled using a spline) on neonatal anthropometrics, using the imputed datasets at selected waist circumference values. The reference waist circumference is 95cm (median of the sample)

|  | Outcome | univariable | | | multivariable | | |
| --- | --- | --- | --- | --- | --- | --- | --- |
| Waist circumference (ref: 95cm) | Birthweight (g) | beta | CI_low | CI_high | beta | CI_low | CI_high |
| 70 |  | -868.1 | -1007.7 | -728.5 | -841.9 | -990.7 | -693.1 |
| 80 |  | -591.1 | -637.7 | -544.6 | -578.5 | -646.9 | -510.1 |
| 90 |  | -202.3 | -222.4 | -182.2 | -199.3 | -250.9 | -147.6 |
| 100 |  | 181.6 | 159.4 | 203.8 | 180.0 | 128.8 | 231.1 |
| 110 |  | 405.2 | 364.7 | 445.6 | 404.7 | 342.2 | 467.1 |
| 120 |  | 364.0 | 240.6 | 487.4 | 370.6 | 237.8 | 503.4 |
| 70 | Head circumference (cm) | -2.8 | -3.3 | -2.3 | -2.7 | -3.3 | -2.2 |
| 80 |  | -1.8 | -2.0 | -1.6 | -1.8 | -2.0 | -1.5 |
| 90 |  | -0.6 | -0.6 | -0.5 | -0.6 | -0.8 | -0.4 |
| 100 |  | 0.5 | 0.4 | 0.6 | 0.5 | 0.3 | 0.7 |
| 110 |  | 1.1 | 0.9 | 1.2 | 1.2 | 0.9 | 1.4 |
| 120 |  | 0.9 | 0.5 | 1.3 | 1.1 | 0.6 | 1.5 |
| 70 | Birth length (cm) | -4.2 | -4.9 | -3.5 | -4.1 | -4.9 | -3.4 |
| 80 |  | -2.6 | -2.8 | -2.4 | -2.5 | -2.9 | -2.2 |
| 90 |  | -0.8 | -0.9 | -0.7 | -0.8 | -1.1 | -0.6 |
| 100 |  | 0.7 | 0.6 | 0.8 | 0.7 | 0.4 | 0.9 |
| 110 |  | 1.5 | 1.3 | 1.7 | 1.4 | 1.1 | 1.8 |
| 120 |  | 1.1 | 0.5 | 1.7 | 1.2 | 0.5 | 1.8 |
| 70 | Ponderal index | -0.2 | -0.3 | -0.1 | -0.2 | -0.3 | -0.1 |
| 80 |  | -0.1 | -0.2 | -0.1 | -0.1 | -0.2 | -0.1 |
| 90 |  | 0.0 | -0.1 | 0.0 | 0.0 | -0.1 | 0.0 |
| 100 |  | 0.0 | 0.0 | 0.1 | 0.0 | 0.0 | 0.1 |
| 110 |  | 0.1 | 0.1 | 0.1 | 0.1 | 0.1 | 0.1 |
| 120 |  | 0.1 | 0.0 | 0.1 | 0.1 | 0.0 | 0.2 |

**Figure S7**: effect of waist circumference (modelled using a spline) on neonatal binary outcomes, using the imputed datasets. The reference waist circumference is 95cm (median of the sample). Shaded band = 95% CI.


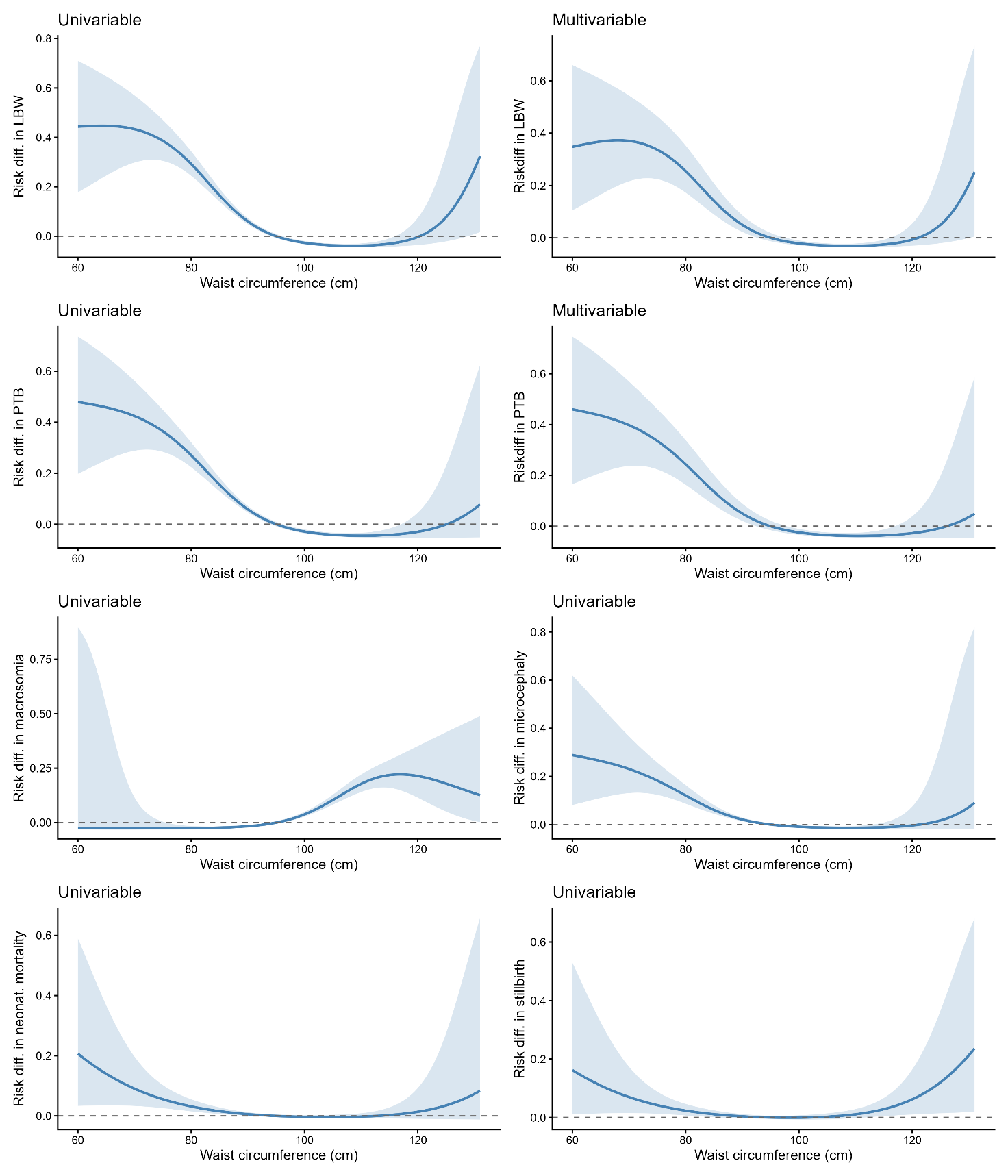


**Table S15**: effect of waist circumference (modelled using a spline) on neonatal binary outcomes, using the imputed datasets at selected waist circumference values. The reference waist circumference is 95cm (median of the sample)

|  | Outcome | univariable | | | multivariable | | |
| --- | --- | --- | --- | --- | --- | --- | --- |
| Waist circumference (ref: 95cm) | LBW (<2,500g) | beta | CI_low | CI_high | beta | CI_low | CI_high |
| 70 |  | 0.43 | 0.30 | 0.57 | 0.37 | 0.22 | 0.54 |
| 80 |  | 0.29 | 0.24 | 0.34 | 0.25 | 0.17 | 0.35 |
| 90 |  | 0.06 | 0.05 | 0.07 | 0.05 | 0.02 | 0.09 |
| 100 |  | 0.00 | -0.01 | 0.01 | -0.02 | -0.03 | -0.01 |
| 110 |  | -0.03 | -0.03 | -0.02 | -0.03 | -0.04 | -0.02 |
| 120 |  | -0.04 | -0.04 | -0.03 | -0.01 | -0.03 | 0.05 |
| 70 | PTB (<37 weeks) | 0.42 | 0.29 | 0.56 | 0.40 | 0.24 | 0.57 |
| 80 |  | 0.27 | 0.22 | 0.32 | 0.24 | 0.16 | 0.34 |
| 90 |  | 0.06 | 0.05 | 0.07 | 0.05 | 0.02 | 0.09 |
| 100 |  | 0.00 | -0.01 | 0.01 | -0.02 | -0.03 | -0.01 |
| 110 |  | -0.03 | -0.04 | -0.02 | -0.04 | -0.04 | -0.03 |
| 120 |  | -0.05 | -0.05 | -0.03 | -0.03 | -0.05 | 0.04 |
| 70 | Macrosomia (>95^th^ perc) | -0.03 | -0.03 | 0.12 |  |  |  |
| 80 |  | -0.03 | -0.03 | -0.02 |  |  |  |
| 90 |  | -0.02 | -0.02 | -0.01 |  |  |  |
| 100 |  | 0.00 | -0.01 | 0.01 |  |  |  |
| 110 |  | 0.04 | 0.03 | 0.05 |  |  |  |
| 120 |  | 0.18 | 0.14 | 0.22 |  |  |  |
| 70 | Microcephaly | 0.23 | 0.13 | 0.37 |  |  |  |
| 80 |  | 0.12 | 0.09 | 0.16 |  |  |  |
| 90 |  | 0.02 | 0.01 | 0.03 |  |  |  |
| 100 |  | 0.00 | 0.00 | 0.01 |  |  |  |
| 110 |  | -0.01 | -0.01 | 0.00 |  |  |  |
| 120 |  | -0.01 | -0.02 | -0.01 |  |  |  |
| 70 | Neonatal mortality | 0.09 | 0.03 | 0.20 |  |  |  |
| 80 |  | 0.03 | 0.02 | 0.06 |  |  |  |
| 90 |  | 0.01 | 0.00 | 0.01 |  |  |  |
| 100 |  | 0.00 | -0.01 | 0.00 |  |  |  |
| 110 |  | 0.00 | -0.01 | 0.01 |  |  |  |
| 120 |  | 0.01 | -0.01 | 0.09 |  |  |  |
| 70 | Stillbirth | 0.07 | 0.02 | 0.17 |  |  |  |
| 80 |  | 0.02 | 0.01 | 0.05 |  |  |  |
| 90 |  | 0.00 | 0.00 | 0.01 |  |  |  |
| 100 |  | 0.00 | -0.01 | 0.01 |  |  |  |
| 110 |  | 0.00 | -0.01 | 0.01 |  |  |  |
| 120 |  | 0.01 | 0.00 | 0.03 |  |  |  |
